# Predicting survival from colorectal cancer histology slides using deep learning: A retrospective multicenter study

**DOI:** 10.1371/journal.pmed.1002730

**Published:** 2019-01-24

**Authors:** Jakob Nikolas Kather, Johannes Krisam, Pornpimol Charoentong, Tom Luedde, Esther Herpel, Cleo-Aron Weis, Timo Gaiser, Alexander Marx, Nektarios A. Valous, Dyke Ferber, Lina Jansen, Constantino Carlos Reyes-Aldasoro, Inka Zörnig, Dirk Jäger, Hermann Brenner, Jenny Chang-Claude, Michael Hoffmeister, Niels Halama

**Affiliations:** 1 Department of Medical Oncology and Internal Medicine VI, National Center for Tumor Diseases, University Hospital Heidelberg, Heidelberg, Germany; 2 German Cancer Consortium (DKTK), Heidelberg, Germany; 3 Applied Tumor Immunity, German Cancer Research Center (DKFZ), Heidelberg, Germany; 4 Division of Gastroenterology, Hepatology and Hepatobiliary Oncology, University Hospital RWTH Aachen, Aachen, Germany; 5 Institute of Medical Biometry and Informatics, University Hospital Heidelberg, Heidelberg, Germany; 6 Institute of Pathology, Heidelberg University, Heidelberg, Germany; 7 Tissue Bank of the National Center for Tumor Diseases (NCT), Heidelberg, Germany; 8 Institute of Pathology, University Medical Center Mannheim, Mannheim, Germany; 9 Division of Clinical Epidemiology and Aging Research, German Cancer Research Center (DKFZ), Heidelberg, Germany; 10 Department of Electrical Engineering, City, University of London, London, United Kingdom; 11 Division of Preventive Oncology, German Cancer Research Center (DKFZ) and National Center for Tumor Diseases (NCT), Heidelberg, Germany; 12 Translational Immunotherapy, German Cancer Research Center (DKFZ), Heidelberg, Germany; University of California San Francisco, UNITED STATES

## Abstract

**Background:**

For virtually every patient with colorectal cancer (CRC), hematoxylin–eosin (HE)–stained tissue slides are available. These images contain quantitative information, which is not routinely used to objectively extract prognostic biomarkers. In the present study, we investigated whether deep convolutional neural networks (CNNs) can extract prognosticators directly from these widely available images.

**Methods and findings:**

We hand-delineated single-tissue regions in 86 CRC tissue slides, yielding more than 100,000 HE image patches, and used these to train a CNN by transfer learning, reaching a nine-class accuracy of >94% in an independent data set of 7,180 images from 25 CRC patients. With this tool, we performed automated tissue decomposition of representative multitissue HE images from 862 HE slides in 500 stage I–IV CRC patients in the The Cancer Genome Atlas (TCGA) cohort, a large international multicenter collection of CRC tissue. Based on the output neuron activations in the CNN, we calculated a “deep stroma score,” which was an independent prognostic factor for overall survival (OS) in a multivariable Cox proportional hazard model (hazard ratio [HR] with 95% confidence interval [CI]: 1.99 [1.27–3.12], *p* = 0.0028), while in the same cohort, manual quantification of stromal areas and a gene expression signature of cancer-associated fibroblasts (CAFs) were only prognostic in specific tumor stages. We validated these findings in an independent cohort of 409 stage I–IV CRC patients from the “Darmkrebs: Chancen der Verhütung durch Screening” (DACHS) study who were recruited between 2003 and 2007 in multiple institutions in Germany. Again, the score was an independent prognostic factor for OS (HR 1.63 [1.14–2.33], *p* = 0.008), CRC-specific OS (HR 2.29 [1.5–3.48], *p* = 0.0004), and relapse-free survival (RFS; HR 1.92 [1.34–2.76], *p* = 0.0004). A prospective validation is required before this biomarker can be implemented in clinical workflows.

**Conclusions:**

In our retrospective study, we show that a CNN can assess the human tumor microenvironment and predict prognosis directly from histopathological images.

## Abstract 2

Jakob Nikolas Kather and colleagues demonstrate the ability of deep convolutional neural networks to extract prognostically relevant information from routine histopathological images collected from colorectal cancer patients.

## Author summary

**Why was this study done?:**

- Colorectal cancer (CRC) is a common disease with a variable clinical course, and there is a high clinical need to more accurately predict the outcome of individual patients.
- For almost every CRC patient, histological slides of tumor tissue are routinely available.
- Deep learning can be used to extract information from very complex images, and we hypothesized that deep learning can predict clinical outcome directly from histological images of CRC.

**What did the researchers do and find?:**

- We trained a deep neural network to identify different tissue types that are abundant in histological images of CRC, especially nontumorous (“stromal”) tissue types.
- We showed that this deep neural network can decompose complex tissue into its constituent parts and that the abundance of each of these tissue parts can be aggregated in a prognostic score.
- In two independent, multicenter patient cohorts, we showed that this score improves survival prediction compared to the Union Internationale Contre le Cancer (UICC) staging system, which is the current state of the art.

**What do these findings mean?:**

- Deep learning is an inexpensive tool to predict the clinical course of CRC patients based on ubiquitously available histological images.
- Prospective validation studies are needed to firmly establish this biomarker for routine clinical use.

## Introduction

Precision oncology depends on stratification of cancer patients into different groups with different tumor genotypes, phenotypes, and clinical outcome. While subjective evaluation of histological slides by highly trained pathologists remains the gold standard for cancer diagnosis and staging, molecular and genetic tests are dominating the field of quantitative biomarkers [1–4].

Pathology slides offer a wealth of information that have for years been quantified by means of digital pathology and classical machine learning techniques [5]. However, few if any digital pathology biomarkers have made their way into the clinic so far, partly because of technological limitations, including complicated image analysis algorithms. Previous work on digital pathology has used computer-based image analysis approaches for cell detection and classification [6], tissue classification [7], nuclei and mitosis detection [8,9], microvessel segmentation [10], and other immunohistochemistry scoring tasks [11] in histopathological images. Machine learning methods can extract prognosticators from such images [12] and have also been used to extract prognosticators from radiological images [13].

Outside of medicine, the advent of convolutional neural networks (CNNs) has revolutionized the image analysis field. Complex visual tasks can be efficiently solved by neural networks that can learn to distinguish objects based on features learned from training data. Applications of CNNs range widely, from speech recognition [14,15], face recognition [16], or traffic sign classification [17] to mastering the Japanese game of Go [18]. We refer to LeCun et al. [19] for an excellent review. In the context of medical imaging, CNNs have been used to classify medical images [20], detect cancer tissue in histopathological images [21], extract prognosticators from tissue microarrays (TMAs) of human solid tumors [22], and classify tumor cell nuclei according to chromatin patterns [23].

While most of these studies have focused on the tumor cells, the stromal compartment—defined as all nontumor components of cancer tissue—is moving into the focus of biomarker research in oncology [24]. In solid tumors such as colorectal cancer (CRC), lymphocytes and fibroblasts profoundly shape the tumor microenvironment and have a significant impact on clinical end points [25,26]. Tumor-infiltrating lymphocytes have been quantified with classical image analysis methods [27,28] and deep learning methods [29], which for some tumor types has been correlated to transcriptomic data [30].

However, the clinical translation of this technological progress is still hampered by two main obstacles: lack of well annotated and abundant data for training CNN models, and validation of these proposed methods in a wide range of clinically relevant situations with heterogeneous real-world data, especially hematoxylin–eosin (HE) images from different institutions.

In the present study, we aimed to fill these gaps in the context of human CRC, a clinically highly relevant disease. We used two large, multicenter collections of histological images and aimed to evaluate the prognostic power of CNNs in these data sets by developing and validating a new prognostic model.

## Methods

### Ethics statement

All experiments were conducted in accordance with the Declaration of Helsinki, the International Ethical Guidelines for Biomedical Research Involving Human Subjects by the Council for International Organizations of Medical Sciences (CIOMS), the Belmont Report, and the US Common Rule. Anonymized archival tissue samples were retrieved from the tissue bank of the National Center for Tumor diseases (NCT; Heidelberg, Germany) in accordance with the regulations of the tissue bank and the approval of the ethics committee of Heidelberg University (tissue bank decision numbers 2152 and 2154, granted to NH and JNK; informed consent was obtained from all patients as part of the NCT tissue bank protocol; ethics board approval S-207/2005, renewed on 20 December 2017). Parts of these samples originated from the DACHS study [31,32]. Another set of tissue samples was provided by the pathology archive at University Medical Center Mannheim (UMM; Heidelberg University, Mannheim, Germany) after approval by the institutional ethics board (Ethics Board II at UMM; decision number 2017-806R-MA, granted to AM and waiving the need for informed consent for this retrospective and fully anonymized analysis of archival samples). HE images from the The Cancer Genome Atlas (TCGA) [33] were downloaded from public repositories at the National Institutes of Health (NIH; USA). These images were randomly drawn from colorectal adenocarcinoma (COAD) and rectal adenocarcinoma (READ) patients.

### Prospective analysis plan

Before starting the study, we planned to train a CNN for multiclass tissue classification in CRC histology, to apply this CNN to histological images of the TCGA cohort and build a predictive score from the output neuron activations. Having done this, we acquired an independent data set (DACHS data set, see below) to validate the predictor. During the peer review process, we added multiple elements of internal and external validation, but we have not changed the predictive model.

### Patient cohorts and data availability

HE-stained human cancer tissue slides from four patient cohorts were used in this study. All images were 224 × 224 pixels (px) and 0.5 μm/px and were normalized with the Macenko method [34]. We used this color normalization method because there were subtle differences in the red and blue hues in the original images, which resulted in a biased classification.

**Fig 1:**
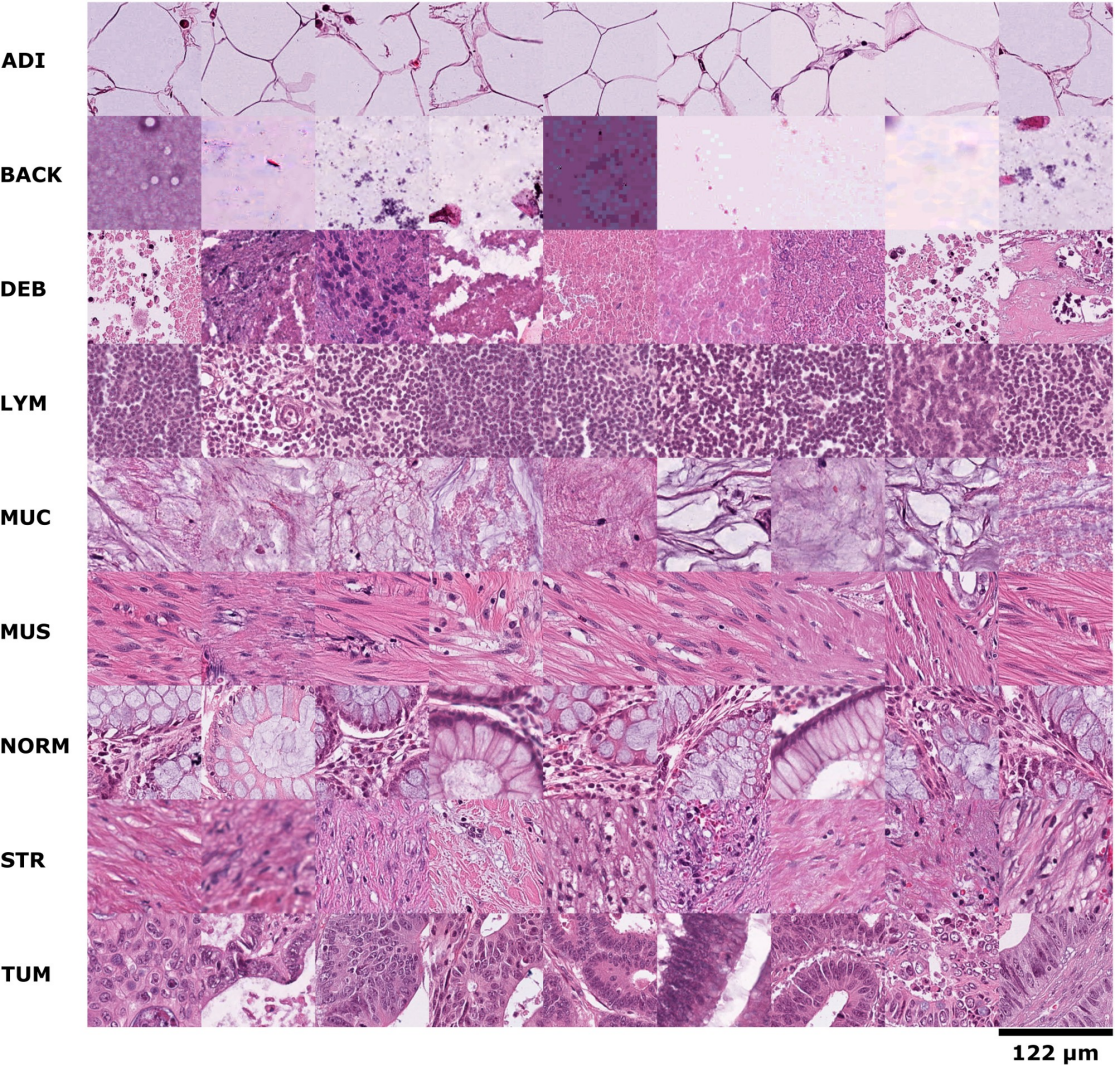
Example images for each of the nine tissue classes represented in the NCT-CRC-HE-100K data set. ADI, adipose tissue; BACK, background; CRC, colorectal cancer; DEB, debris; HE, hematoxylin–eosin; LYM, lymphocytes; MUC, mucus; MUS, smooth muscle; NCT, National Center for Tumor Diseases; NORM, normal colon mucosa; STR, cancer-associated stroma; TUM, colorectal adenocarcinoma epithelium.

First, 86 HE slides of human cancer tissue from the NCT biobank and the UMM pathology archive were used to create a training image set of 100,000 image patches (NCT-CRC-HE-100K, without clinical follow-up data, data available at http://dx.doi.org/10.5281/zenodo.1214456). Representative images of this cohort are shown in Fig 1. We manually delineated regions of pure textures as described before [7] and extracted these nonoverlapping image patches with approximately equal distribution among the following nine tissue classes: adipose tissue, background, debris, lymphocytes, mucus, smooth muscle, normal colon mucosa, cancer-associated stroma, and CRC epithelium. CRC epithelium was only derived from human CRC specimen (primary and metastatic). Normal tissue such as smooth muscle and adipose tissue was mostly derived from CRC surgical specimen, but also from gastrectomy specimen (including upper gastrointestinal smooth muscle) in order to maximize variability in this training set.

Second, 25 HE slides of human CRC tissue from the DACHS study in the NCT biobank were used to create a testing set of 7,180 image patches (CRC-VAL-HE-7K, without clinical follow-up data, data available at http://dx.doi.org/10.5281/zenodo.1214456).

Third, we retrieved 862 HE slides from 500 CRC patients from the TCGA cohort (COAD and READ patients available at http://cancer.digitalslidearchive.net/) [33] with clinical follow-up data and histopathological annotation. The sample size of this cohort was chosen such that the patient number was comparable to the sample sizes in similar studies [35,36]. For the TCGA data set, we used snap-frozen sections only because these are derived from the tissue portions that were also used for molecular analysis. All slides from CRC patients in the TCGA project were manually reviewed, and slides with tissue folds, torn tissue, or other artifacts—as well as slide without any tumor tissue—were excluded. The process of slide selection was done blinded to all other clinicopathological variables, outcome data, or gene expression data. For all TCGA patients in our analysis, we also retrieved gene expression data (available at https://portal.gdc.cancer.gov/) as well as tumor purity estimates as defined by the ABSOLUTE method (described in [37], data available at https://www.synapse.org/#!Synapse:syn3582761). From the digital whole-slide images, we manually extracted regions of 1,500 × 1,500 px at 0.5 μm/px (MPP) from these images. These regions were extracted in such a way that no artifacts were present in the region. The process of extracting the regions was blinded to all clinicopathological data, outcome data, and gene expression data. With matched RNA-seq data from these patients, we calculated a cancer-associated fibroblast (CAF) score as proposed by Isella et al. [38]. The score was computed using the average gene expression levels (RNA-seq) of the CAF signature. The gene lists for the CAF signature is shown in S1 Table. As part of the metadata, manual estimation of total stromal content by pathologists was available. Whenever this information was available for more than one slide per patient, we used the mean in all downstream analyses. This TCGA data set was used to analyze prognostic impact of neural network-based tissue decomposition and deep stroma score (see below) with primary end point being overall survival (OS). A clinicopathological summary of these patients is shown in S2 and S3 Tables. OS by tumor stage for this cohort is shown in S1A Fig. More extensive clinical data on the subjects in this cohort (as required by the TRIPOD checklist) are publicly available via the GDC data portal at https://portal.gdc.cancer.gov/projects/TCGA-COAD and https://portal.gdc.cancer.gov/projects/TCGA-READ. Tissue samples in this cohort were provided by multiple institutions in different countries, which are listed at https://gdc.cancer.gov/resources-tcga-users/tcga-code-tables/tissue-source-site-codes. Subjects with missing outcome data were excluded from the prognostic model, and no imputation was used.

Fourth, we retrieved 409 HE slides from 409 patients in the DACHS cohort [31,32] at the NCT biobank with clinical follow-up data and used this cohort as an independent validation set for the deep stroma score. The primary end point was OS; secondary end points were disease-specific survival (DSS) and relapse-free survival (RFS). The sample size for this cohort was based on tissue availability. A clinicopathological summary of these patients is shown in S4 and S5 Tables. OS by tumor stage for this cohort is shown in S1B Fig. The patients were recruited in multiple hospitals in the Rhine-Neckar region in Germany between 2003 and 2007. More extensive clinical data on the subjects in this cohort, including information about follow-up (as required by the TRIPOD checklist), was described previously [31,32]. Subjects with missing outcome data were excluded from the prognostic model, and no imputation was used.

### Training and testing of neural networks

We used various CNN models, all of which were pretrained on the ImageNet database (www.image-net.org). We replaced the classification layer and trained the whole network with stochastic gradient descent with momentum (SGDM). We evaluated the performance of five different CNN models: a VGG19 model [39], a simpler neural network model, AlexNet [40], a very simple model, SqueezeNet version 1.1 [41], a more complex model, GoogLeNet [42], and a “residual learning” network model, Resnet50 [43]. To gauge the performance of these network architectures, we used the NCT-CRC-HE-100K set and divided it into 70% training set, 15% validation set, and 15% testing set. We trained all networks on a desktop workstation with two NVidia P6000 GPUs with a mini batch size of 360 and a learning rate of 3 × 10^−4^ for eight iterations. We found that all networks with the exception of SqueezeNet achieved >97% classification accuracy in this task. VGG19 had the best performance, with 98.7% accuracy and an acceptable training time (S2 Fig). We therefore used VGG19 for all further experiments. The trained VGG19 model can be downloaded at http://dx.doi.org/10.5281/zenodo.1420524.

In all cases, rotational invariance was achieved through data augmentation with random horizontal and vertical flips of the training images. Images were resized to the neural network input size if necessary. To test the classification accuracy of the neural network with the standard image data sets, images of 224 × 224 px (112 × 112 μm) were fed into the network one at a time.

After neural network training with all 100,000 image patches (which were derived from 86 whole-slide images) in the NCT-CRC-HE-100K set, we assessed tissue classification accuracy in an external validation set: the CRC-VAL-HE-7K data set, which contained 7,180 image patches (derived from 25 whole-slide images). All images in these sets had a size of 224 × 224 px and were presented to the network sequentially.

Next, we applied the network to larger images with heterogeneous tissue composition. We used a sliding window to extract partially overlapping tiles that were presented to the network. The activations of the softmax output layer (layer 46, one output neuron per tissue class, ranging from 0 to 1) were then saved for each image tile. For visualization, each output class was represented by a distinct color. The final color of each pixel in the visualization was the sum of these colors weighted by the output neuron activations at this particular location. To compare different images, the mean activation for each tissue class was used.

### Assessment of neural network training

To assess the quality of neural network training, we employed three separate steps: (1) validation of the classification accuracy in an independent training set, (2) visualization of the class separation based on t-distributed stochastic neighbor embedding (tSNE) [44] of deep layer activations, and (3) DeepDream visualization of deep neuron activations (layer 46 of VGG19, pyramid level 12, iterations 75, scale 1.1, with histogram stretching of the resulting image for optimal visualization).

### Deep stroma score

For each image in the TCGA data set, we used the mean activation of the softmax output neuron for any of the nine output classes in regions of 1,500 × 1500 px (750 × 750 μm). In this set, we sampled one region from the top slide and one region from the bottom slide if both were available. The same procedure was applied to the DACHS set. However, only one slide per patient was available in this set, and we sampled two or three regions from each image depending on image size. In total, 862 image patches were used for the TCGA data set, and 1,349 image patches were used for the DACHS data set (S3 Fig). If several images for one patient were available, the maximum activation was used for each class (max pooling). All image were Macenko-normalized before further analysis [34].

Following tissue decomposition of all images in the training set (TCGA set), we assessed the prognostic performance of each tissue component by using univariable Cox proportional hazard models with continuous predictors (nonthresholded). For the nine classes (adipose, background, debris, lymphocytes, mucus, smooth muscle, normal colon mucosa, cancer-associated stroma, and COAD epithelium), the hazard ratios (HRs) for shorter OS were 1.150, 0.015, 5.967, 1.226, 0.488, 3.761, 0.909, 1.154, and 0.475. Then, optimal cutoffs for the prediction of survival (yes/no) were determined using ROC analysis by selecting the cutoff with the highest Youden index (sensitivity + specificity − 1). If there were multiple optimal cutoffs, the one closer to the median was chosen. These cutoffs were 0.00056, 0.00227, 0.03151, 0.00121, 0.01123, 0.02359, 0.06405, 0.00122, and 0.99961. Next, we combined all tissue components with a HR > 1 into a score by using the following procedure: we counted the number of tissue classes (0 to 5) that were above the optimum Youden threshold for each class, weighted by the HR for each class in order to give more weight to features with higher prognostic power. This HR was derived from a univariable Cox proportional hazard model. Because the resulting metric comprised information from various nontumor (i.e., stromal) components of the tumor, we termed it “deep stromal score.” It should be noted that this notion of “stroma” comprises various nontumor components of the tissue such as desmoplastic stroma, lymphocytes, and adipose tissue. In the TCGA set, the median score value was 8.347, which was subsequently used to stratify patients into high/low in all further analyses. Using this same procedure and the same cutoff in the DACHS data set, 34% of all patients were “high,” and 66% were “low.” Using these dichotomous values, we fitted multivariate Cox proportional hazard models adjusting for the Union Internationale Contre le Cancer (UICC) stage (continuous variable, 1, 2, 3, or 4), sex (male or female), and age in decades (age in years divided by 10) to estimate HRs and corresponding 95% confidence intervals (CIs).

The cutoffs for comparing the prognostic power of different scores were as follows: we used the median for deep stroma score and an optimal threshold (calculated by the Youden method) for CAF score and pathologist annotation. Then, each score was assessed in a dichotomized way in a multivariable Cox proportional hazard model including tumor, node, and metastases (TNM) stage, sex, and age as covariates.

### Summary of the procedures

In summary, we systematically tested five neural network models for a transfer-learning–based classification task in 100,000 histological image patches. VGG19 was the best model in an internal and an external testing set (S2 Fig, details on the model in S6 Table). We then used this model to extract tissue characteristics from complex histological images with clinical annotation and combined these data in a “deep stroma score.” This score was evaluated in two large patient cohorts with a total of 909 patients. A flowchart of the full procedure is shown in S3 Fig. Our study complies with the TRIPOD statement [45] as declared in S7 Table.

### Software

All statistical analyses were done in R unless otherwise noted (R version 3.4.0) using the following libraries: survminer, survival, ggfortify, ggplot2, OptimalCutpoints (and their respective dependencies). *p* < 0.05 was considered statistically significant; *p* ≥ 0.05 was considered not significant (n.s.). JASP version 0.8.5.1 was used for descriptive statistics. Neural network training and deployment was done in Matlab R2018a on two standard desktop workstations with two Nvidia Quadro P6000 GPUs and a Nvidia Titan Xp GPU, respectively. Our source codes are available at http://dx.doi.org/10.5281/zenodo.1471616.

## Results

### CNNs can learn morphological features in histological images

**Fig 2:**
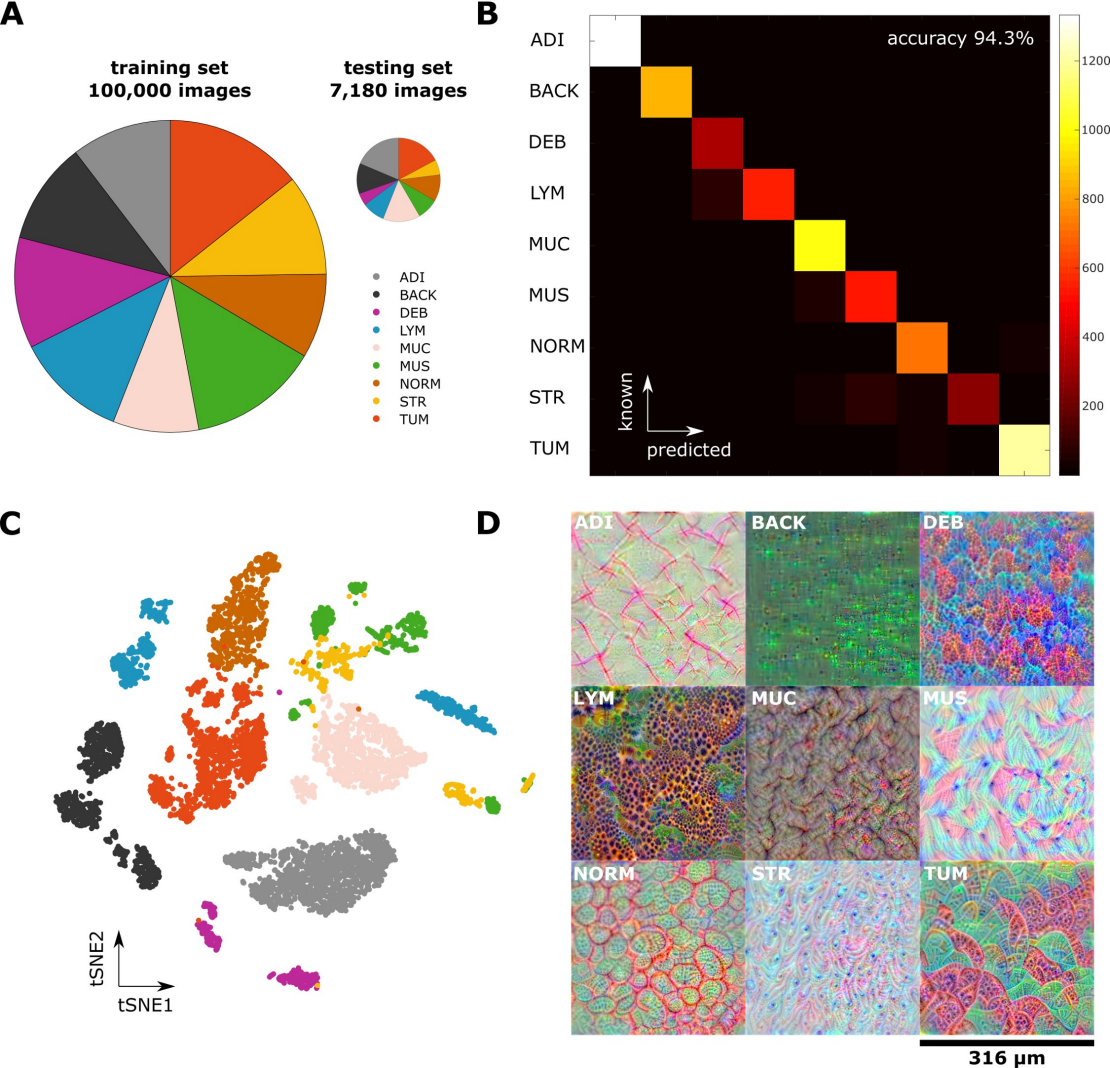
A CNN learns robust representations of histological images and attains high classification accuracy. (A) A nine-class training set containing 100,000 unique images and a testing set of 7,180 unique images. Classes are adipose, background, debris, lymphocytes, mucus, smooth muscle, normal mucosa, stroma, cancer epithelium. Pie area is proportional to sample number. (B) Confusion matrix of the CNN-based classification; overall accuracy is 94%. (C) tSNE of the testing set based on deep layer activations of the trained CNN. Tissue classes naturally aggregate in separate clusters, with close proximity of the TUM and NORM cluster and the MUS and STR cluster, respectively. (D) Deep dream visualization of the spatial patterns represented in the trained CNN. For all tissue classes, the network has learned to visually discern key features. For example, LYM are composed of tightly collected small round cells, and NORM is composed of glands in an even distribution pattern. ADI, adipose tissue; BACK, background; CNN, convolutional neural network; DEB, debris; LYM, lymphocytes; MUC, mucus; MUS, smooth muscle; NORM, normal mucosa; STR, stroma; tSNE, t-distributed stochastic neighbor embedding; TUM, cancer epithelium.

We used our NCT-HE-100K data set of 100,000 histological images to train a VGG19 CNN model and tested the classification performance in an independent set of 7,180 images from different patients (CRC-VAL-HE-7K, Fig 2A). The overall nine-class accuracy was close to 99% in an internal testing set (S2 Fig) and 94.3% in an external testing set. A high accuracy was obtained in all tissue classes (Fig 2B). Most misclassifications arose between the classes muscle and stroma as well as between lymphocytes and debris/necrosis (Fig 2B). This misclassification was expected because muscle and stroma share a fibrous architecture and necrosis is often infiltrated by inflammatory cells (Fig 1). In a similar multiclass problem in CRC image analysis, previous methods have attained well below 90% accuracy [7]. We visualized the internal representations of tissue classes by using tSNE on deep layer activations and saw a near perfect separation of the classes in the testing set (Fig 2C). This shows that the CNN learns image features that allow a separation of nine tissue classes.

**Fig 3:**
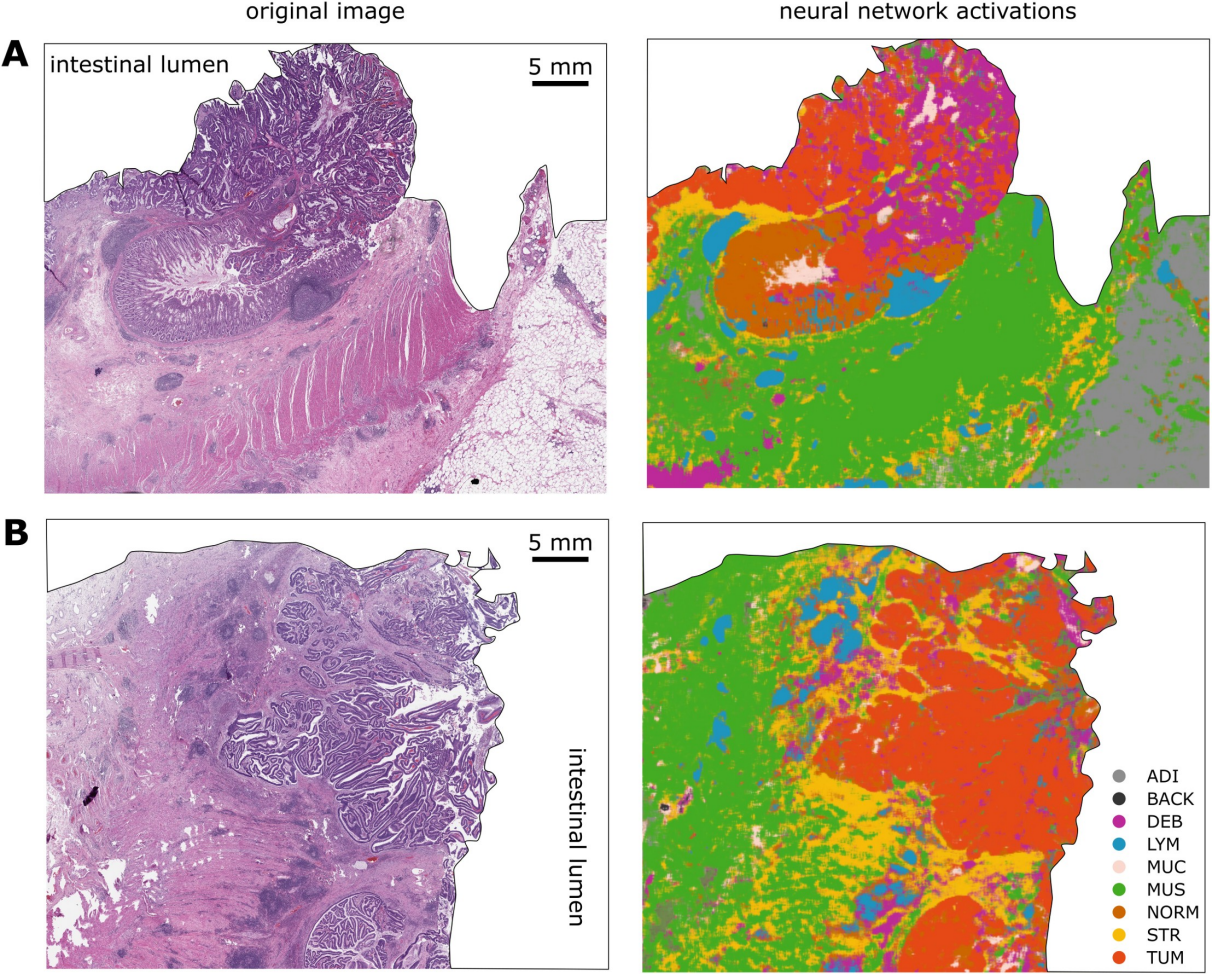
A CNN can segment histopathological whole-slide images. The neural network classifier was used to classify real-world images from the DACHS cohort. (A) and (B) show two representative example images. Left: original HE image; right: classification map. Even fine structures are recognized by the neural network even in regions of suboptimal tissue quality. Only the tissue is shown in this example, and because the tissue does not occupy a rectangular area on the pathology slide, the whole-slide image was manually segmented by an observer trained in pathology to show only tissue without background for better clarity (background is white). ADI, adipose tissue; BACK, background; CNN, convolutional neural network; DACHS, Darmkrebs: Chancen der Verhütung durch Screening; DEB, debris; HE, hematoxylin–eosin; LYM, lymphocyte aggregates; MUC, mucus; MUS, muscle; NORM, normal mucosa; STR, stroma; TUM, tumor epithelium.

Next, we visualized the morphological features learned by the network using a DeepDream approach, which to our knowledge has not been used in the context of histological imaging before. As can be seen in Fig 2D, the tissue structures that were learned by the network are well understandable for human vision: examples are loosely aligned tissue fibers in muscle and stroma, the regular textures present in normal colonic mucosa, and the more irregular texture present in colorectal carcinoma epithelium. We applied the neural network to larger images, with examples shown in S4A–S4M Fig, and to whole-slide images, two representative images of which are shown in Fig 3A and 3B. Especially in the whole-slide images, the neural network achieved a high classification accuracy that matches human perception. For two major tissue classes, tumor and stroma, we visualized deep layer activations using tSNE (S5A and S5B Fig). We saw that, within these classes, similar phenotypes were grouped together, and different phenotypes were located are at a larger distance from one another. For stroma, dense stroma and loose stroma formed separate clusters (S5A and S5B Fig). For tumor, well differentiated and poorly differentiated tumor parts each formed a separate cluster (S5C and S5D Fig).

Based on these data, we conclude that CNNs develop internal representations of different tissue classes and that they are capable of solving multiclass tissue classification problems better than the previous state of the art [7]. Our data provide evidence that training a CNN model with a large data set results in excellent performance, exceeding the state of the art for histological tissue classification. Detailed performance statistics of our model are available in S6 Fig and S8 Table.

### CNNs can decompose complex tissue

**Fig 4:**
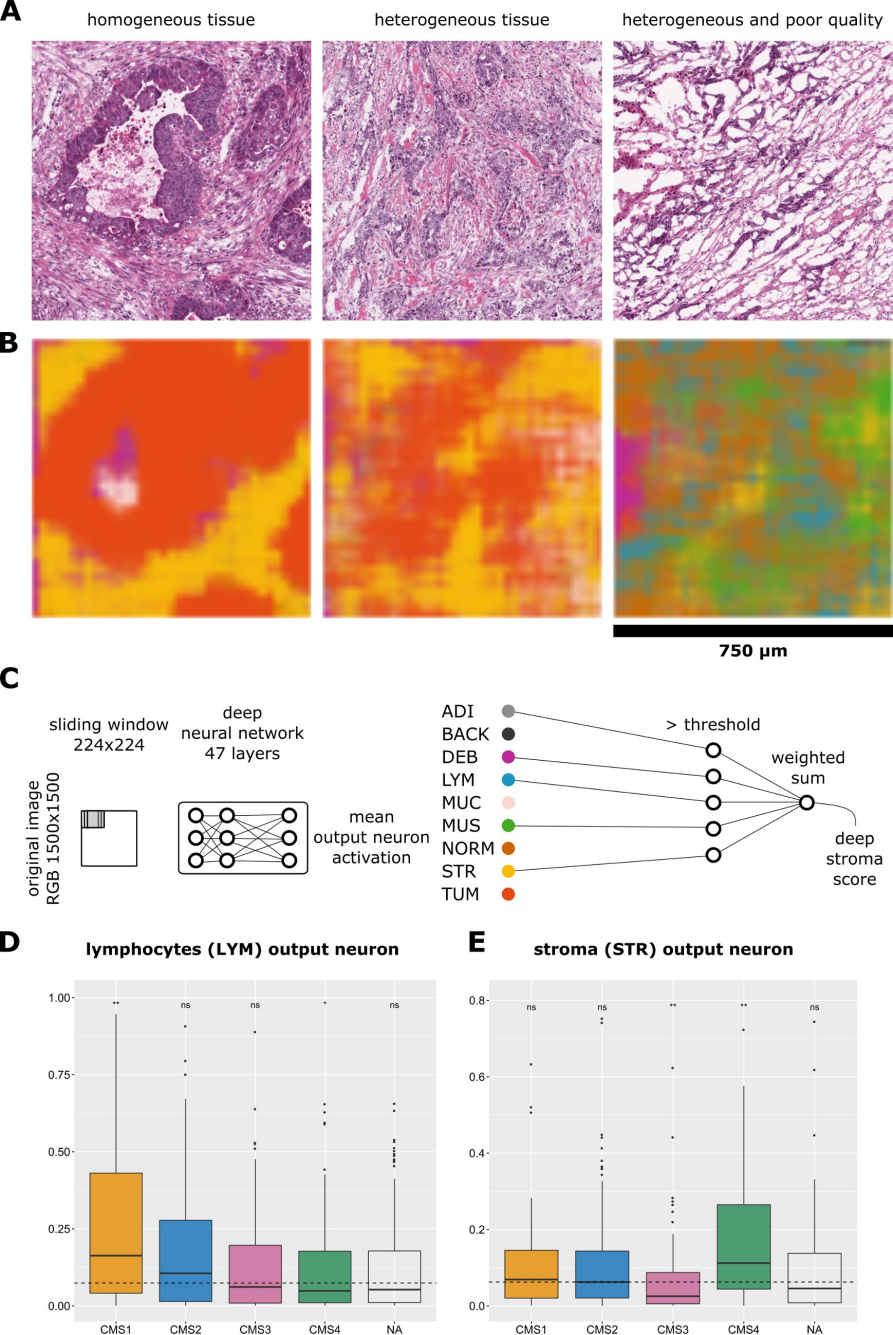
Prognostication of CRC outcome by a deep stroma score. (A) HE images in the TCGA cohort had heterogeneous texture, and some had poor quality. Image size is 1,500 × 1,500 px, and regions were classified with a sliding window of 224 × 224 px. (B) Neural network activations corresponding to the images shown in panel A are visualized. Even in the poor-quality case, tissue structures are recognized by the network. (C) A deep stroma score based on neural network activations is defined as the weighted sum of stromal tissue classes that are above threshold. (D, E) Mean output layer activation for lymphocytes and stroma separated by CMS. Activation of (D) lymphocytes and (E) stroma were assessed in images from 425 patients from the TCGA cohort. As expected, CMS1 highly activated the lymphocyte output neuron, while CMS4 highly activates the stroma output neuron. *p ≤ 0.05, **p ≤ 0.01; ns > 0.05; two-tailed *t* test for each group versus all samples. The dashed line marks the mean of all samples against which *t* test was performed. The line within each box marks the median of that group, the full box contains all samples between the 25th and the 75th percentile, and the vertical lines extend to the smallest and largest nonoutlier value (R ggplot2 geom_boxplot convention). CMS, consensus molecular subtype; CRC, colorectal cancer; HE, hematoxylin–eosin; LYM, lymphocytes; NA, not available; ns, not significant; px, pixels; STR, stroma; TCGA, The Cancer Genome Atlas.

Based on the finding that a CNN could classify tissue components in histological images, we next assessed whether this approach can be used to extract prognostically relevant information from images. To this end, we used a large data set of clinically annotated HE whole-slide images from 500 patients from the TCGA cohort. These images came from various institutions and were derived from snap-frozen tissue sections with varying quality (Fig 4A). Using partially overlapping tiles, we classified the tissue in these complex images, yielding plausible neural network activation maps (Fig 4B).

CRC can be separated into four distinct consensus molecular subtypes (CMSs) [46] that are correlated to different cell populations in the tumor microenvironment [47]. For all patients with available RNA-seq data, we calculated the CMS as described previously [46]. It is known that CMS1 tumors are highly infiltrated by lymphocytes and CMS4 tumors contain abundant desmoplastic stroma. CNN tissue decomposition yielded compatible results: activation of the lymphocyte output neuron was significantly (*p* < 0.001) increased in CMS1 tumors (Fig 4D) compared to all tumors. Activation of the stroma output neuron was significantly (*p* < 0.001) increased in CMS4 tumors (Fig 4E) compared to all tumors. We conclude that CNNs can decompose complex tissue parts and consistently identify tissue components that are known to be present in specific molecular subtypes of CRC.

### CNNs can extract prognosticators from HE images

**Fig 5:**
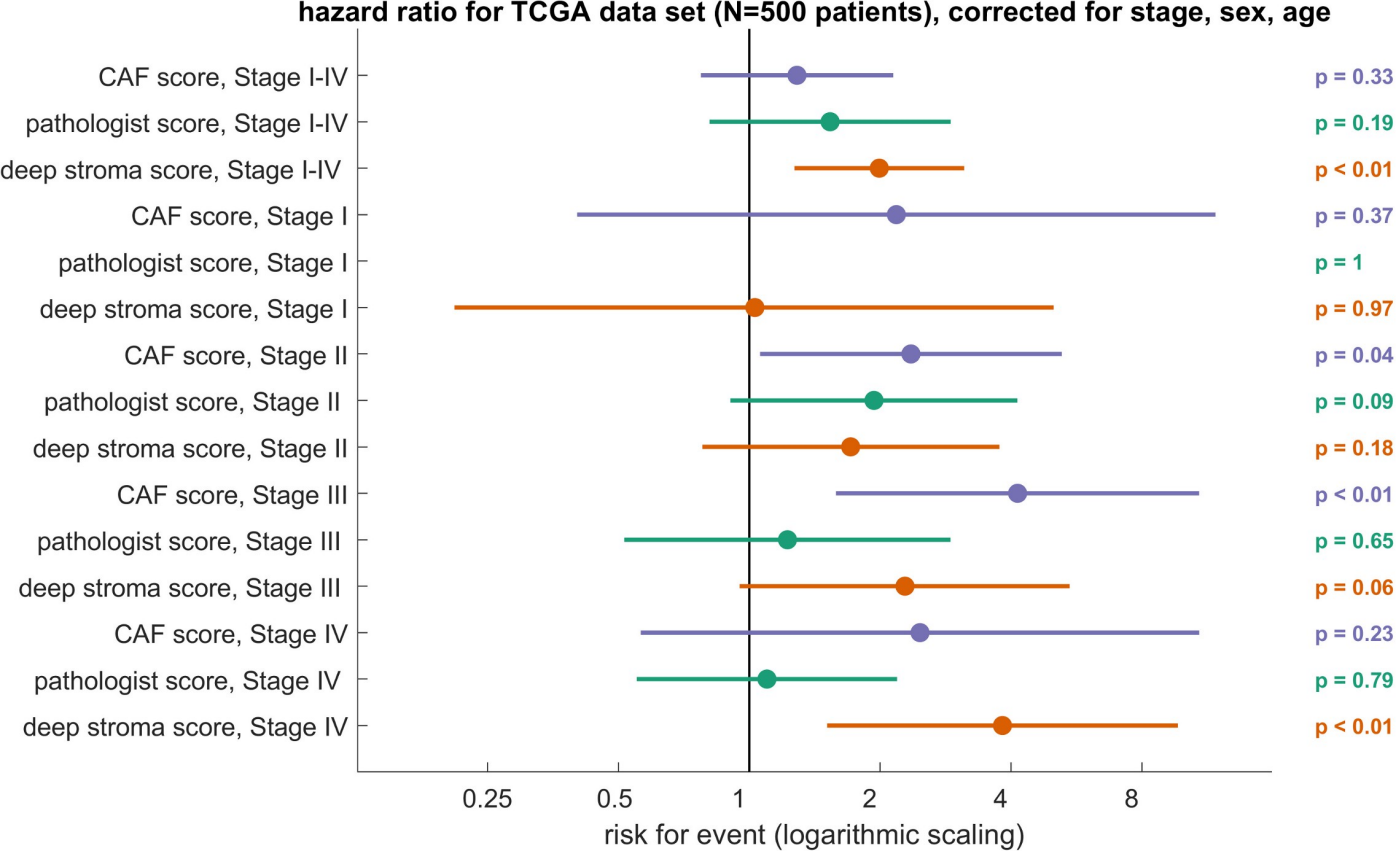
Deep stroma score is an independent prognosticator for shorter OS in the TCGA cohort. HRs with 95% CI in multivariable Cox models including cancer stage (I–IV), sex, and age for a CAF gene expression score, pathologist’s manual quantification of stromal percentage as provided in the TCGA metadata and the deep stroma score. The deep stroma score was binarized into high/low at the median. The other scores (CAF, pathologist) were binarized at an optimal threshold (optimal Youden index). Only the deep stroma score was significantly associated with prognosis in the whole cohort (stage I–IV). The horizontal axis is scaled logarithmically (log 10). CAF, cancer-associated fibroblast; CI, confidence interval; HR, hazard ratio; OS, overall survival; TCGA, The Cancer Genome Atlas.

Having thus confirmed that CNN extract plausible data from complex images with mixed tissues, we next investigated whether activation of the class output neurons carries prognostic information. We fitted univariable Cox proportional hazard models to each output class and found that higher activation of five of nine classes was correlated to a poor outcome (adipose tissue: HR = 1.150 [n.s.]; debris: HR = 5.967 [*p* = 0.004]; lymphocytes: HR = 1.226 [n.s.]; muscle: HR = 3.761 [*p* = 0.025]; stroma: HR = 1.154 [n.s.]). Based on these findings, we investigated whether a “deep stroma score” that combined all of these features was an independent prognostic factor for survival. This deep stroma score was a combination of multiple nontumor components of the tissue as quantified by the output neuron activation of a CNN. Indeed, in the TCGA data set, the deep stroma score was a prognostic factor for shorter OS in a univariate (HR 2.12 [1.38–3.23], *p* = 0.001) and an independent prognostic factor in a multivariate Cox proportional hazard model (HR 1.99 [1.27–3.12], *p* = 0.0028, Fig 5) with UICC stage, gender, and age as covariates (Fig 5). We hypothesized that there might be tumor-stage–specific differences in the prognostic power and therefore calculated the multivariable Cox model for each tumor stage. Indeed, the HR for shorter OS increased with increasing tumor stage as shown in Fig 5. We conclude that a deep stroma score based on tissue decomposition by a CNN is an independent prognostic factor in CRC patients with considerable prognostic power, especially in advanced tumor stages (UICC 4).

### Neural network assessment of the stromal compartment compared to gold standard methods

Having shown that a deep stroma score carries prognostic power, we compared this approach to current gold standard methods to assess the stromal component of CRC. Two such standard methods are manual estimation of stromal percentage in HE sections by pathologists and a gene expression signature of CAFs. In the TCGA cohort, manual annotation was available as part of the metadata. Also, gene expression data were available, from which we calculated a CAF score as proposed by Isella et al. [38]. The CAF score quantifies fibroblasts only, while the pathologist’s annotation quantifies areas of desmoplastic stroma. Both measures are known to be associated with survival in CRC and other types of cancer [48–51]. It should be noted, however, that these measures capture different information than our deep stroma score, which is a combination of multiple nontumor components, including but not limited to desmoplastic stroma.

For each score, we calculated the HR for shorter OS in a multivariate Cox proportional hazard model, using CAF signature, pathologist annotation, and deep stroma score, respectively, along with UICC stage, gender, and age as covariates. The CAF signature was independently prognostic for survival in stage II (HR = 2.35 [1.06–5.23], *p* = 0.036) and stage III (HR = 4.14 [1.58–10.82], *p* = 0.0038) tumors, while the pathologist’s annotation was not prognostic in any tumor stage. The deep stroma score was an independent prognostic factor in stage IV tumors (Fig 5). Also, the deep stroma score was highly significantly prognostic of survival in the full cohort of all tumor stages (HR = 1.99 [1.27–3.12], *p* = 0.0028), while CAF score and pathologist annotation were not (Fig 5).

Deep stroma score values were not significantly correlated to CAF score or pathologist annotation (*p* > 0.05). However, the stroma component of the deep stroma score itself was moderately correlated to the CAF score (Pearson’s correlation coefficient is 0.26, *p* < 0.001). This is higher than the correlation between pathologist annotation and CAF score (correlation coefficient 0.20, *p* < 0.001), suggesting that the neural network is at least as good as pathologists at detecting the stromal component as reflected in gene expression analysis. We also compared the output of the CNN tumor output neuron to tumor purity estimates and found that there was a poor correlation (correlation coefficient 0.069, *p* = 0.14). Together, these findings suggest that the deep neural network is not a good extractor for tumor-cell–related components but is an efficient extractor of stromal components.

Furthermore, we compared the performance of our model to the UICC TNM stage which is the gold standard for prognostication in CRC. As shown in S1 Fig, TNM stage is a well-established predictor of survival, and by itself, it is a better predictor than the deep stroma score alone. However, as the multivariable analysis shows (Fig 5), the deep stroma score remains a significant predictor of survival in a multivariable risk model that includes TNM stage.

### Deep stroma score generalizes to an independent validation cohort from a different institution

**Fig 6:**
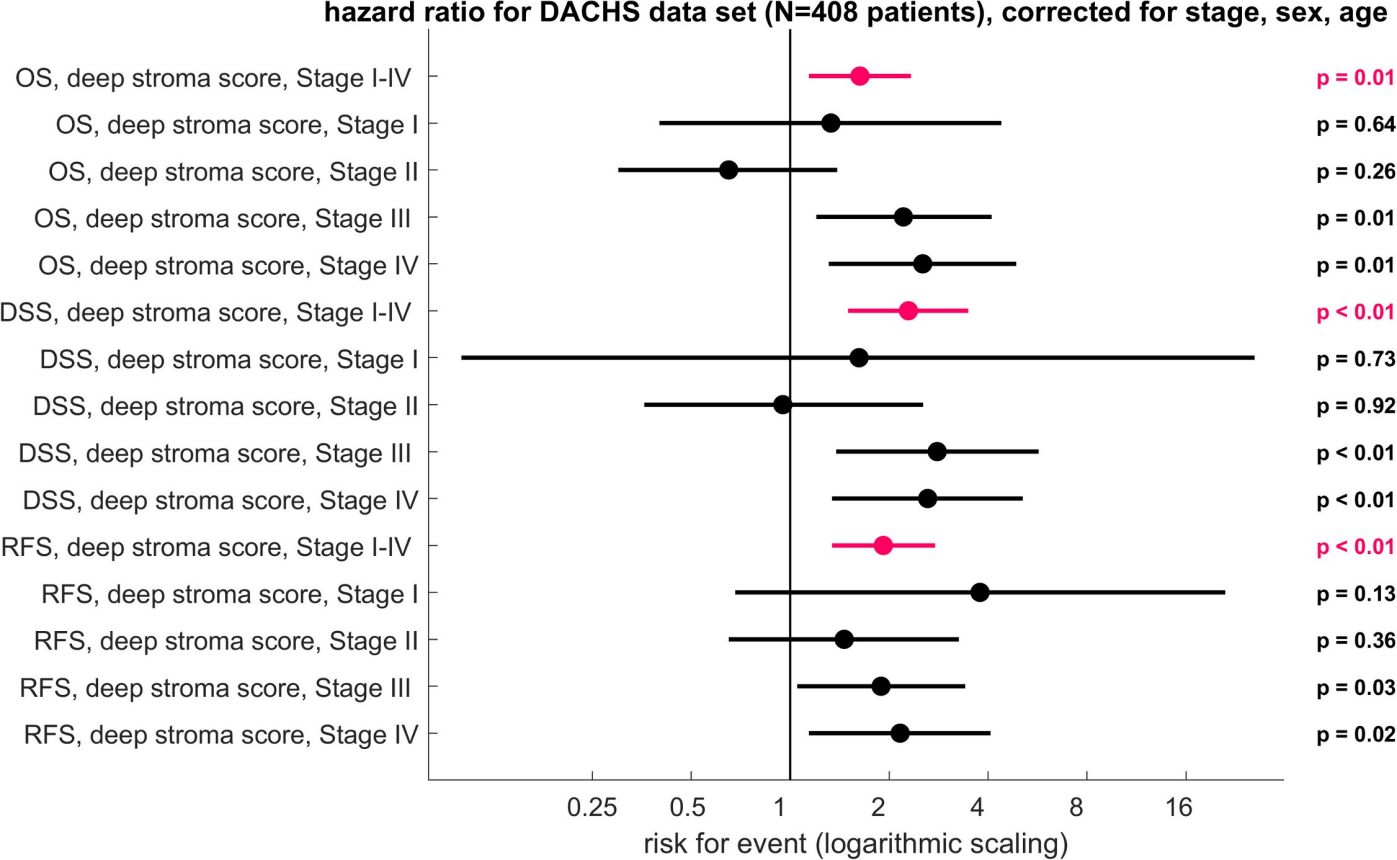
Deep stroma score applied to the validation data set (DACHS cohort). HRs with 95% CI in multivariable Cox models including cancer stage (I–IV), sex, and age for the deep stroma score. HR for OS, DSS, and RFS are plotted. The deep stroma score was stratified into high/low at the median of the training set. In this validation cohort, the deep stroma score was an independent prognostic factor over all stages and within stage III and stage IV tumors. The horizontal axis is scaled logarithmically (log 10). CI, confidence interval; DACHS, Darmkrebs: Chancen der Verhütung durch Screening; DSS, disease-specific survival; HR, hazard ratio; OS, overall survival; RFS, relapse-free survival.

Having shown that the deep stroma score carries prognostic information, we validated this approach in an independent patient cohort. Complex biomarkers often fail when applied to validation cohorts from different institutions, partly because of high variability in tissue samples. We used HE-stained slides from formalin-fixed paraffin-embedded (FFPE) tissue from 409 CRC patients in the DACHS study, a large multicenter study in southwest Germany [31]. We calculated the deep stroma score in these patients, using exactly the same cutoff values as found in the TCGA cohort. We performed multivariate analysis for OS, disease-specific (CRC-specific) survival (DSS), and RFS. Corresponding to the results from the TCGA cohort, we found that the deep stroma score was a highly significant prognosticator for OS (HR 1.63 [1.14–2.33], *p* = 0.008), DSS (HR 2.29 [1.5–3.48], *p* = 0.0004), and RFS (HR 1.92 [1.34–2.76], *p* = 0.0004) in these patients (Fig 6). This was independent of CRC stage, sex, or age (Fig 6). Regarding different UICC stages of the tumor, this effect was n.s. in stage 1 and 2 but was highly significant in stage 3 and 4 cancer (multivariable-adjusted CRC-specific survival for UICC stage 1 cancer: HR 1.62 [n.s.]; stage 2: HR 0.95 [n.s.]; stage 3: HR 2.8 [*p* = 0.0044]; stage 4: HR 2.62 [*p* = 0.0047]; Fig 6). Again, we could show that the deep stroma score is an independent prognostic factor with strong prognostic power, especially in advanced tumors.

## Discussion

In this study, we show that stromal microenvironment patterns as analyzed by a CNN are prognostic of OS in a training set of 500 patients and prognostic of OS, DSS, and RFS in an independent validation set of 409 patients, independently of tumor stage, sex, and age. We show that the deep stroma score significantly extends the UICC TNM system, which is the current state of the art and uses much more comprehensive data. Recently, Danielsen et al. have proposed a biomarker that uses digital image analysis to predict prognosis in stage II CRC [52]. The biomarker we present in this study is an independent prognostic factor in advanced CRC (stage III and IV), thereby complementing these recent findings.

Interpretation of complex images by deep CNNs is presently transforming many domains in medical imaging, but clinical translation of this technology is still in its infancy. One reason for this delay is that CNNs per se need huge annotated training data sets that are not readily available in the context of histopathology. Another reason is that neural network–based risk assessment needs to be validated in clinically characterized validation cohorts. In the present study, we addressed both of these difficulties: we assembled a large data set of 100,000 histological image patches, by far exceeding previous comparable publicly available data sets. Furthermore, we analyzed two large patient cohorts to establish and validate a CNN-based assessment as a prognostic biomarker in human CRC. With this approach, we could indeed show that CNNs are highly capable of classifying histological image patches and of segmenting histological images of complex tissue architecture. Furthermore, we could show that neural-network–based tissue decomposition can be used to calibrate a deep stroma score that is prognostic of OS in a large cohort of patients. Validating this approach in a separate cohort, we confirmed the prognostic power of this approach. Thus, we present a novel biomarker that can be incorporated into existing clinical workflows because it only relies on HE images, which are widely available.

In CRC, the stromal compartment has been shown to carry prognostically valuable information that can be retrieved by subjective pathological evaluation, classical digital pathology approaches, or via genomic and proteomic studies. However, to our knowledge, the prognostic information present in the stromal compartment has not yet been mined via deep learning techniques. Thus, our method constitutes a precedent case for accessing hidden information in the stromal compartment of CRC in an objective and reproducible way.

Our study is a proof of concept that can be the basis for prospective clinical evaluation. Immediate areas of interest would be to identify high-risk patients with advanced cancer who might benefit from more intense treatment. In a digital pathology workflow, our method could be used to automatically detect CRC tumor tissue and—for one or more regions within the tumor—calculate the deep stromal score. This would not replace, but rather augment and accelerate, the pathologist’s evaluation of tissue slides, at the same time making it more objective and reproducible.

As in all studies that employ deep learning methods, the question arises what the deep stroma score represents exactly. The CNN quantifies the different components of nontumorous tissue, combining them into one number: the deep stroma score. Thus, at first glance, it acts as a proportion predictor of the various tissue classes. However, having a softmax layer as output, the CNN can also quantify mixtures of different tissues. An example is an image patch that has a 30% resemblance to desmoplastic stroma but a 70% resemblance to tumor epithelium. This type of problem is apparent in Fig 4A (middle and right panel)—in these cases, the tissue is highly mixed, and for a human observer, it is not easily possible to assign proportions of the different tissue classes. Our approach also differs from gene expression–based methods to estimate the stromal contribution to the total tissue mass, which infers stromal proportion from bulk sequencing data of heterogeneous tissue. Compared to this, a major advantage of our deep learning method is the ubiquitous availability of HE slides—these are available for every cancer patient, and scanning and analyzing them is not very costly. Also, our approach is reproducible: If presented with the same image twice, the algorithm will output the same result. These points make this new approach well suited for a clinical application.

As a retrospective study, this study needs to be validated prospectively before routine clinical use. Another limitation is that, in our study, a blinded observer manually extracted tumor regions from histological whole-slide images. This manual step could be replaced in a fully automatic workflow.

As part of our study, we provide openly accessible sets of annotated histological images, their size exceeding currently available datasets by a factor of 20 [7]. This is important because progress in deep learning is driven by the availability of large annotated collections of training data, and such data are sparse in the field of digital pathology. Thus, our method and our data sets can be used as a benchmark for future trials.

## Supporting information

S1 Fig OS in the TCGA and DACHS cohort, stratified by UICC stage I, II, III, and IV (cleanstage). Log rank *p* < 0.0001 for panels A and B. DACHS, Darmkrebs: Chancen der Verhütung durch Screening; OS, overall survival; TCGA, The Cancer Genome Atlas; UICC, Union Internationale Contre le Cancer.
(TIF)

S2 Fig Comparison of three CNN architectures. The image data set with 100,000 images in nine classes was divided into 70% training set, 15% validation set, and 15% testing set. Five different networks (alexnet, googlenet, resnet50, squeezenet, and vgg19) were trained on this data set. VGG19 achieved the best classification accuracy (98.7%) in this internal test set and was used for all subsequent experiments. Squeezenet had a classification accuracy <<50% and is not shown. CNN, convolutional neural network.
(TIF)

S3 Fig Flowchart of the study. (A) First, we used an image set of 100,000 histological images to find the best neural network model among three candidates. VGG19 achieved the best classification accuracy in an internal test set. (B) We then trained a VGG19 model on the full set of 100,000 images and tested the prediction accuracy in an external test set of >7,000 images. Still, classification accuracy was excellent. (C) We then used this trained model to extract stroma features from clinically annotated slides from 409 patients in the DACHS cohort. We assessed the predictive performance in images from 500 patients in the TCGA cohort. We found that this yields a statistically significant, independent prognostic factor for CRC. CRC, colorectal cancer; DACHS, Darmkrebs: Chancen der Verhütung durch Screening; TCGA, The Cancer Genome Atlas.
(TIF)

S4 Fig Softmax layer activations for larger images in the DACHS cohort. (A–M) Representative images from this data set; left: HE after color normalization; right: output neuron activations (softmax layer [layer 46]). DACHS, Darmkrebs: Chancen der Verhütung durch Screening; HE, hematoxylin–eosin.
(TIF)

S5 Fig Clustering of stromal and tumoral phenotypes. Deep neuron activation (fc7 layer in the VGG19 model) from the training set NCT-CRC-HE-100K were extracted for all images in the classes STR and TUM. These activation vectors were visualized using tSNE. Representative images from four regions (top, bottom, left, right) are shown. (A) tSNE for class STR, four regions are colored. (B) Example images from these regions. (C) tSNE for class TUM, (D) example images for these images. Both for STR and TUM, closely related tissue phenotypes are close in the tSNE representation. For example, in the lower panel of B, dense stroma image patches cluster together, while in the top and left panel, loose stroma clusters together. For TUM, well differentiated glandular adenocarcinoma tissue is enriched in the left region in panel D, while poorly differentiated homogeneous tissue is enriched in the top panel in panel D. STR, stroma; tSNE, t-distributed stochastic neighbor embedding; TUM, cancer epithelium.
(TIF)

S6 Fig ROC curves of classification performance in an external validation set. The external validation set consisted of 7,180 images in nine tissue classes (CRC-VAL-HE-7K data set) and was randomly split into k = 25 subsets. The classifier was applied to each of these subsets. For each tissue class and each subset, the ROC curve is plotted, and the AUC is given as median with the 5th and 95th percentile of their distribution. AUC, area under the curve; CI, confidence interval; ROC, Receiver Operating Characteristic.
(TIF)

S1 Table Genes used for the CAF signature, established by Isella et al. (35). CAF, cancer-associated fibroblast.
(DOCX)

S2 Table Categorical variables of the TCGA cohort. (DOCX)

S3 Table Continuous variables of the TCGA cohort. (DOCX)

S4 Table Categorical variables of the DACHS cohort. (DOCX)

S5 Table Continuous variables of the DACHS cohort. (DOCX)

S6 Table All layers in the final modified VGG19 CNN model. (DOCX)

S7 Table TRIPOD compliance statement. (DOCX)

S8 Table Statistics for each tissue class in an external validation set. AUC, sensitivity, specificity, PPV, and NPV are shown as median with the 5th and 95th percentile of their distribution based on k = 25 random splits of the external validation set as shown in S6 Fig. AUC, area under the curve; CI, confidence interval; NPV, negative predictive value; PPV, positive predictive value.
(DOCX)

## Abbreviations

CAF: cancer-associated fibroblast
CI: confidence interval
CIOMS: the Council for International Organizations of Medical Sciences
CMS: consensus molecular subtype
CNN: convolutional neural network
COAD: colorectal adenocarcinoma
CRC: colorectal cancer
DACHS: Darmkrebs: Chancen der Verhütung durch Screening
DSS: disease-specific survival
FFPE: formalin-fixed paraffin-embedded
HE: hematoxylin–eosin
HR: hazard ratio
NCT: National Center for Tumor Diseases
NIH: National Institutes of Health
n.s.: not significant
OS: overall survival
px: pixels
READ: rectal adenocarcinoma
RFS: relapse-free survival
SGDM: stochastic gradient descent with momentum
TCGA: The Cancer Genome Atlas
TMA: tissue microarray
TNM: tumor, node, and metastases
tSNE: t-distributed stochastic neighbor embedding
UICC: Union Internationale Contre le Cancer
UMM: University Medical Center Mannheim
