# Supplementary figures and images for: Predicting survival from colorectal cancer histology slides using deep learning: A retrospective multicenter study
Source: PLoS Med. 2019 Jan 24;16(1):e1002730. doi: 10.1371/journal.pmed.1002730 (PMC6345440; doi:10.1371/journal.pmed.1002730)

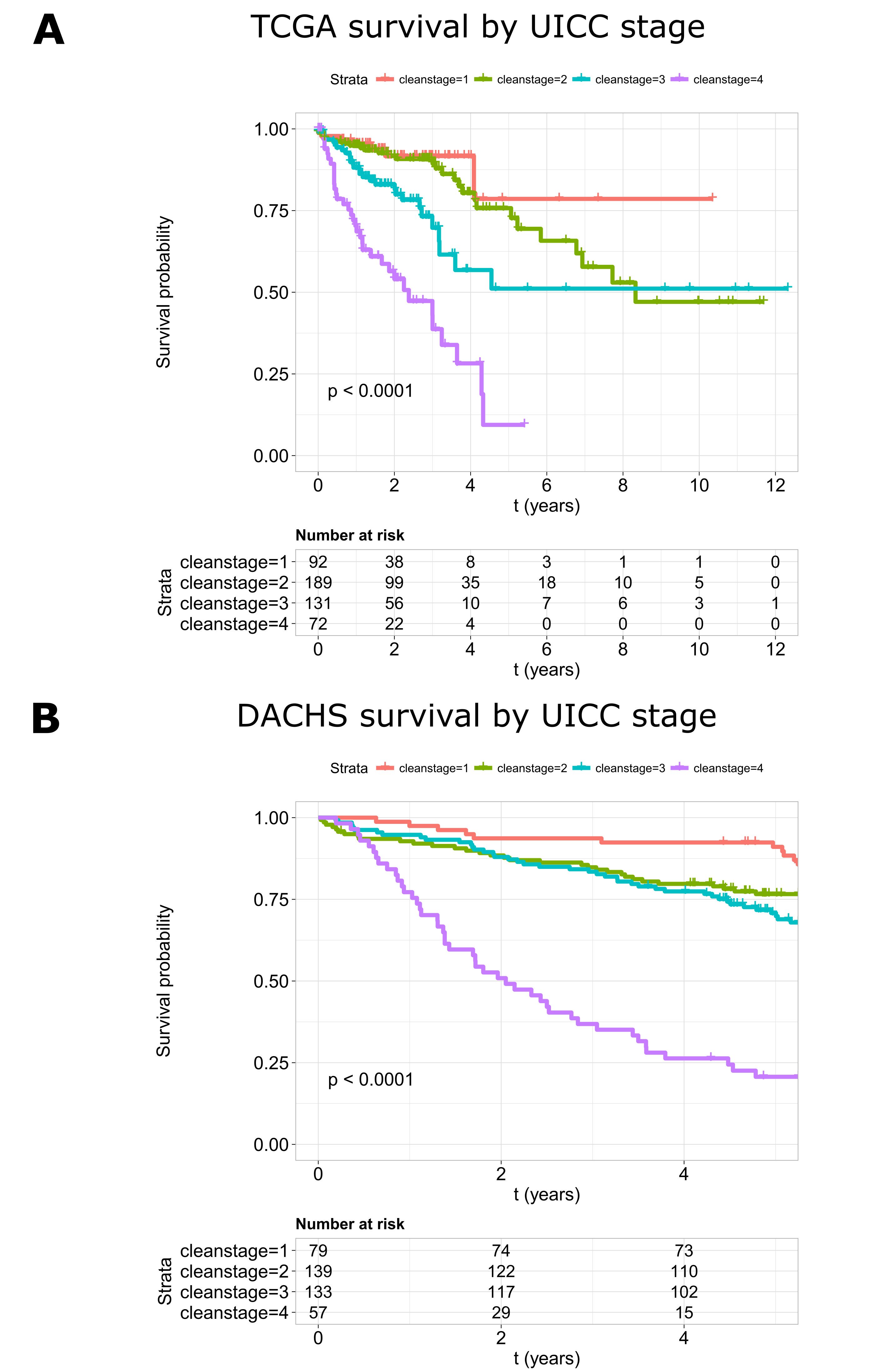

Supplement: S1 Fig — Log rank p < 0.0001 for panels A and B. DACHS, Darmkrebs: Chancen der Verhütung durch Screening; OS, overall survival; TCGA, The Cancer Genome Atlas; UICC, Union Internationale Contre le Cancer. (TIF) [file pmed.1002730.s001.tif]

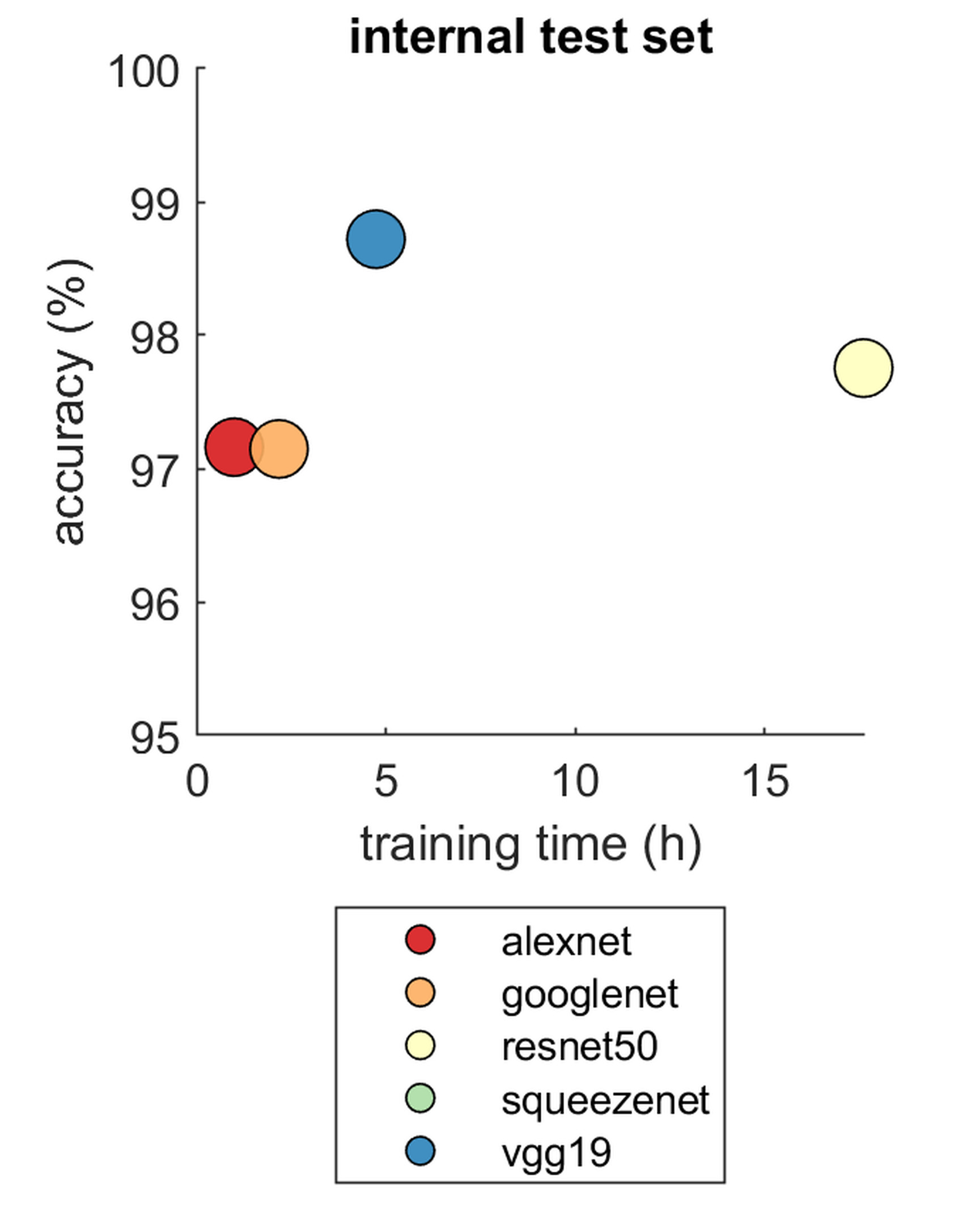

Supplement: S2 Fig — The image data set with 100,000 images in nine classes was divided into 70% training set, 15% validation set, and 15% testing set. Five different networks (alexnet, googlenet, resnet50, squeezenet, and vgg19) were trained on this data set. VGG19 achieved the best classification accuracy (98.7%) in this internal test set and was used for all subsequent experiments. Squeezenet had a classification accuracy <<50% and is not shown. CNN, convolutional neural network. (TIF) [file pmed.1002730.s002.tif]

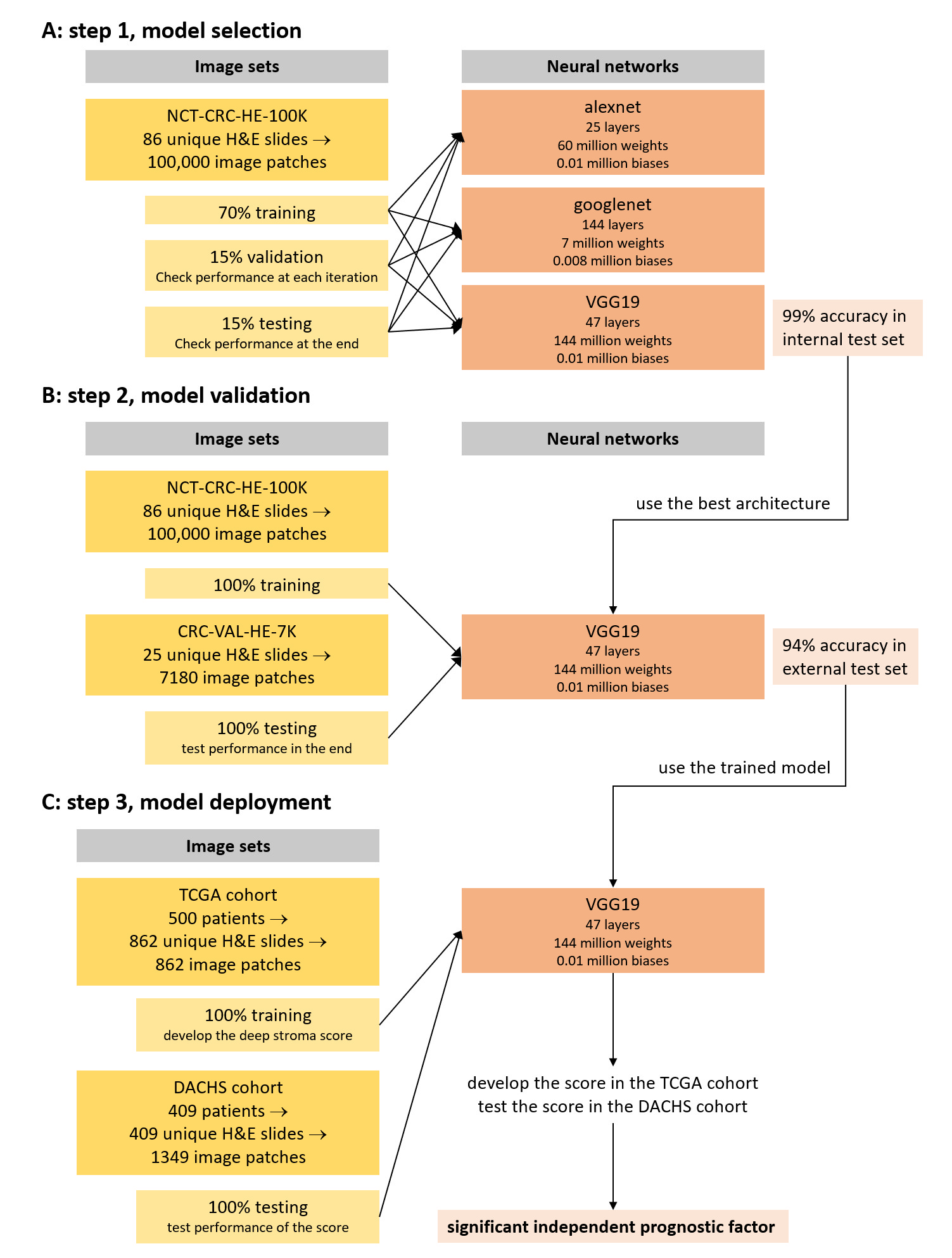

Supplement: S3 Fig — (A) First, we used an image set of 100,000 histological images to find the best neural network model among three candidates. VGG19 achieved the best classification accuracy in an internal test set. (B) We then trained a VGG19 model on the full set of 100,000 images and tested the prediction accuracy in an external test set of >7,000 images. Still, classification accuracy was excellent. (C) We then used this trained model to extract stroma features from clinically annotated slides from 409 patients in the DACHS cohort. We assessed the predictive performance in images from 500 patients in the TCGA cohort. We found that this yields a statistically significant, independent prognostic factor for CRC. CRC, colorectal cancer; DACHS, Darmkrebs: Chancen der Verhütung durch Screening; TCGA, The Cancer Genome Atlas. (TIF) [file pmed.1002730.s003.tif]

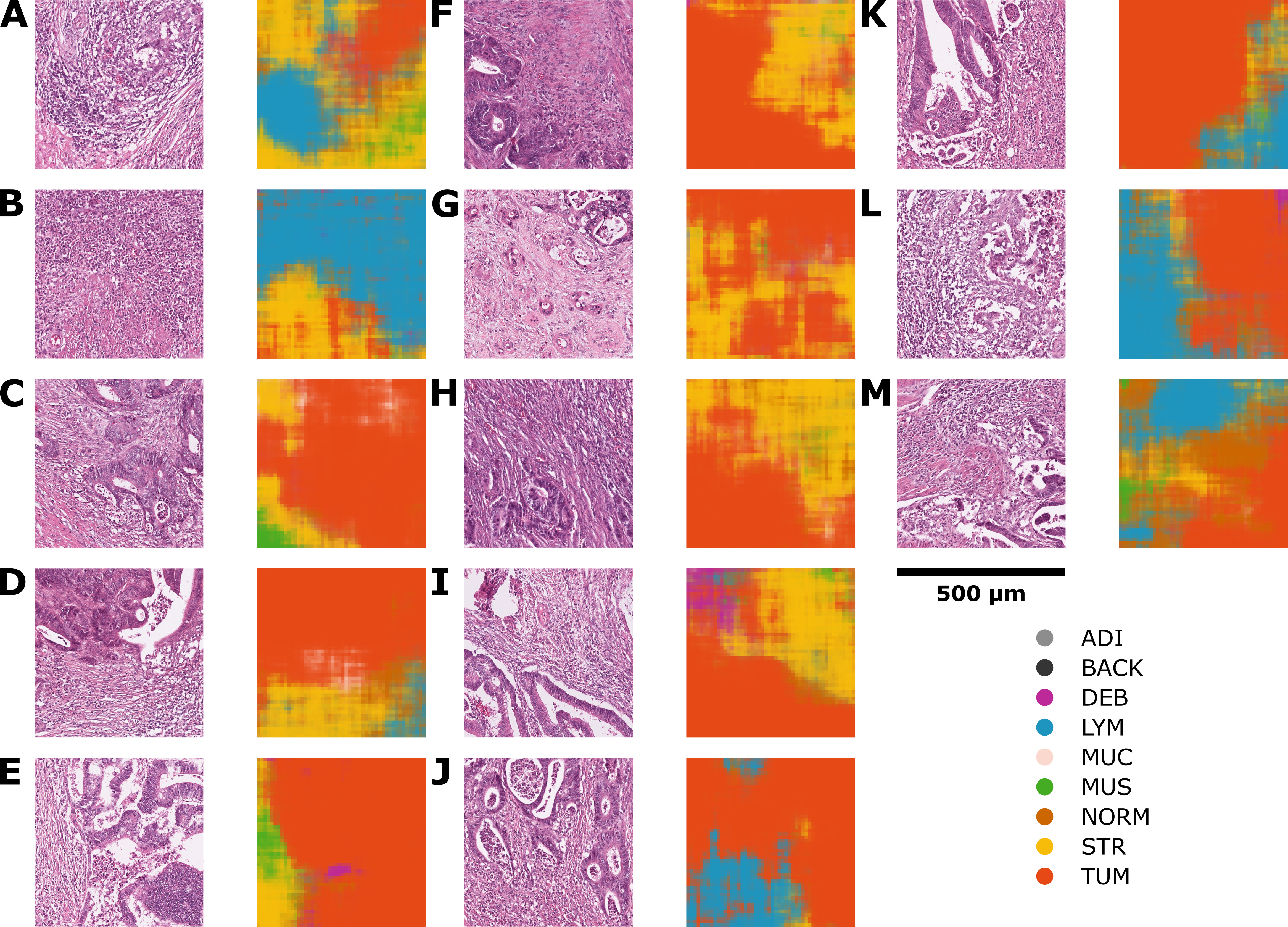

Supplement: S4 Fig — (A–M) Representative images from this data set; left: HE after color normalization; right: output neuron activations (softmax layer [layer 46]). DACHS, Darmkrebs: Chancen der Verhütung durch Screening; HE, hematoxylin–eosin. (TIF) [file pmed.1002730.s004.tif]

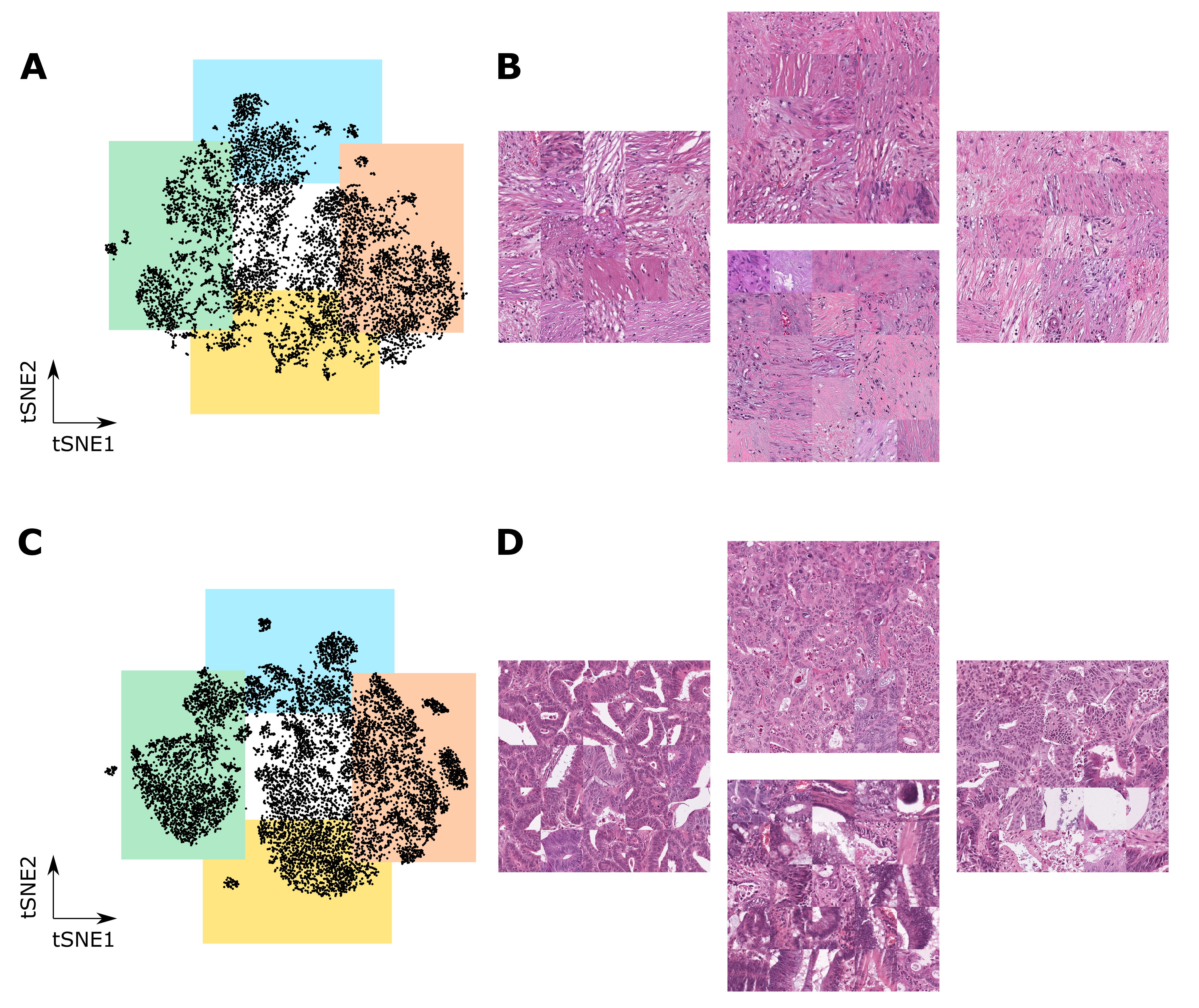

Supplement: S5 Fig — Deep neuron activation (fc7 layer in the VGG19 model) from the training set NCT-CRC-HE-100K were extracted for all images in the classes STR and TUM. These activation vectors were visualized using tSNE. Representative images from four regions (top, bottom, left, right) are shown. (A) tSNE for class STR, four regions are colored. (B) Example images from these regions. (C) tSNE for class TUM, (D) example images for these images. Both for STR and TUM, closely related tissue phenotypes are close in the tSNE representation. For example, in the lower panel of B, dense stroma image patches cluster together, while in the top and left panel, loose stroma clusters together. For TUM, well differentiated glandular adenocarcinoma tissue is enriched in the left region in panel D, while poorly differentiated homogeneous tissue is enriched in the top panel in panel D. STR, stroma; tSNE, t-distributed stochastic neighbor embedding; TUM, cancer epithelium. (TIF) [file pmed.1002730.s005.tif]

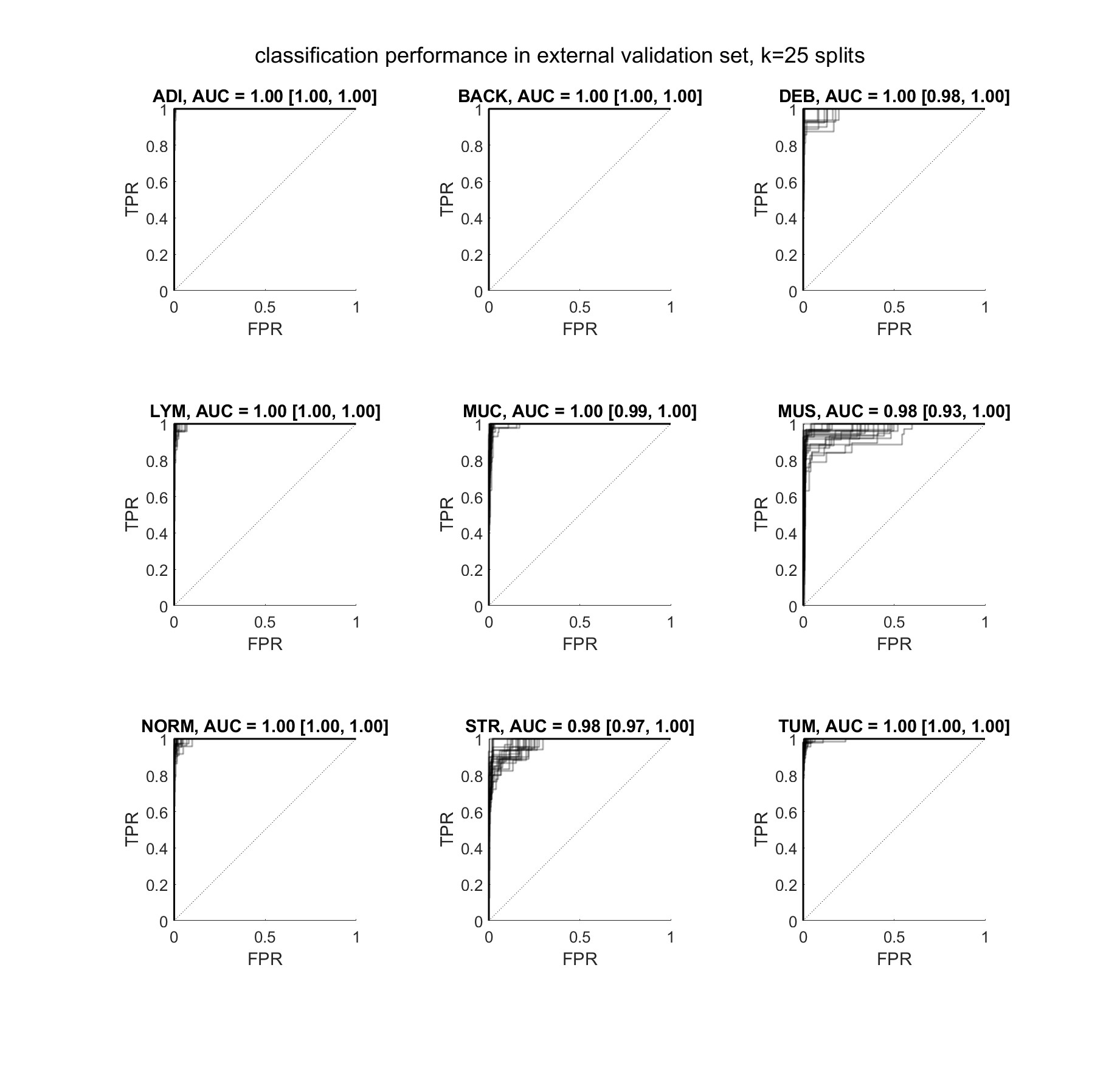

Supplement: S6 Fig — The external validation set consisted of 7,180 images in nine tissue classes (CRC-VAL-HE-7K data set) and was randomly split into k = 25 subsets. The classifier was applied to each of these subsets. For each tissue class and each subset, the ROC curve is plotted, and the AUC is given as median with the 5th and 95th percentile of their distribution. AUC, area under the curve; CI, confidence interval; ROC, Receiver Operating Characteristic. (TIF) [file pmed.1002730.s006.tif]
