# Supplementary material for: Predicting survival from colorectal cancer histology slides using deep learning: A retrospective multicenter study
Source: PLoS Med. 2019 Jan 24;16(1):e1002730. doi: 10.1371/journal.pmed.1002730 (PMC6345440; doi:10.1371/journal.pmed.1002730)
Supplement: S1 Table — CAF, cancer-associated fibroblast. (DOCX) [file pmed.1002730.s007.docx]

| INHBA | PTN | LOC100132116 | MEG3 | CCL2 | LOXL2 |
| --- | --- | --- | --- | --- | --- |
| GPC6 | BNC2 | PDPN | RAB31 | GUCY1B3 | EFEMP1 |
| TGFB3 | ADAM12 | C1R | SGCE | GLT8D2 | COL12A1 |
| HGF | DPT | CPXM1 | SFRP2 | THY1 | C1S |
| FBN1 | CDR1 | FHL1 | LMOD1 | NCAM1 | DDR2 |
| ANTXR1 | CYGB | TRO | CCDC80 | HTRA3 | CCL11 |
| EDIL3 | GALNTL2 | LHFP | GGT5 | ACAN | UNC5C |
| WISP1 | ABCA9 | PDE1A | ADAMTSL3 | EVC | MOXD1 |
| SGCD | MAB21L2 | C9orf47 | CCL13 | MFAP5 | SCARA5 |
| PLXDC1 | PLAT | FST | SFRP1 | PDGFRA | BOC |
| DZIP1 | PLEKHH2 | COL14A1 | DIO2 | SLC26A10 | COL8A1 |
| ISLR | COL11A1 | TNFAIP6 | F2RL2 | PCDH18 | OLFML2B |
| KCNJ8 | KIAA1755 | 4-Sep | FOXF1 | MRGPRF | ASPN |
| CH25H | FOXL1 | FNDC1 | WNT2B | PTGIS | WBSCR17 |
| FBN2 | ITGA11 | SFRP4 | CPZ | LOX | MGC24103 |
| WNT2 | FIBIN | RASSF8 | SMO | MMP3 | MEIS1 |
| WNT5A | CHRDL1 | RGMA | TNFSF11 | EPHA3 | KCNE4 |
| PAMR1 | HHIP | GLI2 | PPAPDC1A | SMARCD3 | GRP |
| LRRC17 | LRRN4CL | COLEC12 | TFPI2 | SPOCK1 | TSHZ3 |
| PTGER3 | ADAMDEC1 | MMP19 | PCDH7 | ABCA6 | TMEM119 |
| MASP1 | GAS1 | CYP7B1 | CCDC102B | NEXN | FAM65C |
| OLFML1 | DSEL | CD302 | ADH1B | OGN |  |
