# Supplementary material for: Predicting survival from colorectal cancer histology slides using deep learning: A retrospective multicenter study
Source: PLoS Med. 2019 Jan 24;16(1):e1002730. doi: 10.1371/journal.pmed.1002730 (PMC6345440; doi:10.1371/journal.pmed.1002730)
Supplement: S2 Table — (DOCX) [file pmed.1002730.s008.docx]

| \| Tumor site \| \| Frequency \| \| Percent \| \| --- \| --- \| --- \| --- \| --- \| \| colon adenocarcinoma \|  \| 316 \|  \| 63.2 \| \| colon mucinous adenocarcinoma \|  \| 48 \|  \| 9.6 \| \| rectal adenocarcinoma \|  \| 115 \|  \| 23.0 \| \| rectal mucinous adenocarcinoma \|  \| 11 \|  \| 2.2 \| \| Missing \|  \| 10 \|  \| 2.0 \| \| Total \|  \| 500 \|  \| 100.0 \| | \| MSI status \| \| Frequency \| \| Percent \| \| \| --- \| --- \| --- \| --- \| --- \| --- \| \| MSI-H \|  \| 66 \|  \| 13.2 \|  \| \| MSI-L \|  \| 71 \|  \| 14.2 \|  \| \| MSS \|  \| 347 \|  \| 69.4 \|  \| \| Missing \|  \| 16 \|  \| 3.2 \|  \| \| Total \|  \| 500 \|  \| 100.0 \|  \| |
| --- | --- | --- | --- | --- | --- | --- | --- | --- | --- | --- | --- | --- | --- | --- | --- | --- | --- | --- | --- | --- | --- | --- | --- | --- | --- | --- | --- | --- | --- | --- | --- | --- | --- | --- | --- | --- | --- | --- | --- | --- | --- | --- | --- | --- | --- | --- | --- | --- | --- | --- | --- | --- | --- | --- | --- | --- | --- | --- | --- | --- | --- | --- | --- | --- | --- | --- | --- | --- | --- | --- | --- | --- |
| \| UICC stage \| \| Frequency \| \| Percent \| \| \| --- \| --- \| --- \| --- \| --- \| --- \| \| 1 \|  \| 92 \|  \| 18.4 \|  \| \| 2 \|  \| 189 \|  \| 37.8 \|  \| \| 3 \|  \| 131 \|  \| 26.2 \|  \| \| 4 \|  \| 72 \|  \| 14.4 \|  \| \| Missing \|  \| 16 \|  \| 3.2 \|  \| \| Total \|  \| 500 \|  \| 100.0 \|  \| | \| CMS \| \| Frequency \| \| Percent \| \| --- \| --- \| --- \| --- \| --- \| \| CMS1 \|  \| 57 \|  \| 11.4 \| \| CMS2 \|  \| 145 \|  \| 29.0 \| \| CMS3 \|  \| 68 \|  \| 13.6 \| \| CMS4 \|  \| 106 \|  \| 21.2 \| \| Missing \|  \| 124 \|  \| 24.8 \| \| Total \|  \| 500 \|  \| 100.0 \| |
| \| Sex \| \| Frequency \| \| Percent \| \| \| --- \| --- \| --- \| --- \| --- \| --- \| \| female \|  \| 234 \|  \| 46.8 \|  \| \| male \|  \| 266 \|  \| 53.2 \|  \| \| Missing \|  \| 0 \|  \| 0.0 \|  \| \| Total \|  \| 500 \|  \| 100.0 \|  \| | \| OS event \| \|  \| Frequency \| \| Percent \| \| \| --- \| --- \| --- \| --- \| --- \| --- \| --- \| \| 0 \|  \|  \| 394 \|  \| 78.8 \|  \| \| 1 \|  \|  \| 106 \|  \| 21.2 \|  \| \| Missing \|  \|  \| 0 \|  \| 0.0 \|  \| \| Total \|  \|  \| 500 \|  \| 100.0 \|  \| |
