# Supplementary material for: Predicting survival from colorectal cancer histology slides using deep learning: A retrospective multicenter study
Source: PLoS Med. 2019 Jan 24;16(1):e1002730. doi: 10.1371/journal.pmed.1002730 (PMC6345440; doi:10.1371/journal.pmed.1002730)
Supplement: S3 Table — (DOCX) [file pmed.1002730.s009.docx]

|  | | | | | | | | | |
| --- | --- | --- | --- | --- | --- | --- | --- | --- | --- |
|  | | Age (years) | | CAF score | | Percent stromal cells (Pathologist) | | Follow up (days) | |
| Valid |  | 499 |  | 500 |  | 500 |  | 500 |  |
| Missing |  | 1 |  | 0 |  | 0 |  | 0 |  |
| Mean |  | 66.21 |  | 2.150 |  | 16.35 |  | 817.9 |  |
| Std. Deviation |  | 12.84 |  | 0.7111 |  | 12.28 |  | 767.1 |  |
| Minimum |  | 31.00 |  | 0.5281 |  | 0.000 |  | 0.000 |  |
| Maximum |  | 90.00 |  | 4.417 |  | 76.00 |  | 4502 |  |
|  | | | | | | | | | |
