# Supplementary material for: Predicting survival from colorectal cancer histology slides using deep learning: A retrospective multicenter study
Source: PLoS Med. 2019 Jan 24;16(1):e1002730. doi: 10.1371/journal.pmed.1002730 (PMC6345440; doi:10.1371/journal.pmed.1002730)
Supplement: S4 Table — (DOCX) [file pmed.1002730.s010.docx]

| \| Tumor site \| \| Frequency \| \| Percent \| \| \| --- \| --- \| --- \| --- \| --- \| --- \| \| colon \|  \| 236 \|  \| 57.7 \|  \| \| rectosigmoid \|  \| 28 \|  \| 6.8 \|  \| \| rectum \|  \| 144 \|  \| 35.2 \|  \| \| Missing \|  \| 1 \|  \| 0.2 \|  \| \| Total \|  \| 409 \|  \| 100.0 \|  \| | \| MSI \| \| Frequency \| \| Percent \| \| \| --- \| --- \| --- \| --- \| --- \| --- \| \| 0 \|  \| 350 \|  \| 85.6 \|  \| \| 1 \|  \| 28 \|  \| 6.8 \|  \| \| Missing \|  \| 31 \|  \| 7.6 \|  \| \| Total \|  \| 409 \|  \| 100.0 \|  \| |
| --- | --- | --- | --- | --- | --- | --- | --- | --- | --- | --- | --- | --- | --- | --- | --- | --- | --- | --- | --- | --- | --- | --- | --- | --- | --- | --- | --- | --- | --- | --- | --- | --- | --- | --- | --- | --- | --- | --- | --- | --- | --- | --- | --- | --- | --- | --- | --- | --- | --- | --- | --- | --- | --- | --- | --- | --- | --- | --- | --- | --- | --- | --- | --- | --- | --- | --- | --- |
| \| UICC stage \| \| Frequency \| \| Percent \| \| \| --- \| --- \| --- \| --- \| --- \| --- \| \| 1 \|  \| 79 \|  \| 19.3 \|  \| \| 2 \|  \| 139 \|  \| 34.0 \|  \| \| 3 \|  \| 133 \|  \| 32.5 \|  \| \| 4 \|  \| 57 \|  \| 13.9 \|  \| \| Missing \|  \| 1 \|  \| 0.2 \|  \| \| Total \|  \| 409 \|  \| 100.0 \|  \| |  |
| \| Sex \| \| Frequency \| \| Percent \| \| --- \| --- \| --- \| --- \| --- \| \| 1 \|  \| 177 \|  \| 43.3 \| \| 2 \|  \| 231 \|  \| 56.5 \| \| Missing \|  \| 1 \|  \| 0.2 \| \| Total \|  \| 409 \|  \| 100.0 \| | \| OS event \| \| Frequency \| \| Percent \| \| \| --- \| --- \| --- \| --- \| --- \| --- \| \| 0 \|  \| 279 \|  \| 68.2 \|  \| \| 1 \|  \| 129 \|  \| 31.5 \|  \| \| Missing \|  \| 1 \|  \| 0.2 \|  \| \| Total \|  \| 409 \|  \| 100.0 \|  \| |
| \| RFS event \| \| Frequency \| \| Percent \| \| \| --- \| --- \| --- \| --- \| --- \| --- \| \| 0 \|  \| 281 \|  \| 68.7 \|  \| \| 1 \|  \| 127 \|  \| 31.1 \|  \| \| Missing \|  \| 1 \|  \| 0.2 \|  \| \| Total \|  \| 409 \|  \| 100.0 \|  \| | \| DFS event \| \| Frequency \| \| Percent \| \| \| --- \| --- \| --- \| --- \| --- \| --- \| \| 0 \|  \| 313 \|  \| 76.5 \|  \| \| 1 \|  \| 93 \|  \| 22.7 \|  \| \| Missing \|  \| 3 \|  \| 0.7 \|  \| \| Total \|  \| 409 \|  \| 100.0 \|  \| |
