# Supplementary material for: Predicting survival from colorectal cancer histology slides using deep learning: A retrospective multicenter study
Source: PLoS Med. 2019 Jan 24;16(1):e1002730. doi: 10.1371/journal.pmed.1002730 (PMC6345440; doi:10.1371/journal.pmed.1002730)
Supplement: S5 Table — (DOCX) [file pmed.1002730.s011.docx]

|  | | Age (years) | | Follow up OS (days) | | Follow up RFS (days) | |
| --- | --- | --- | --- | --- | --- | --- | --- |
| Valid |  | 408 |  | 408 |  | 405 |  |
| Missing |  | 1 |  | 1 |  | 4 |  |
| Mean |  | 68.03 |  | 1543 |  | 1396 |  |
| Std. Deviation |  | 10.85 |  | 587.3 |  | 655.1 |  |
| Minimum |  | 33.00 |  | 10.00 |  | 10.00 |  |
| Maximum |  | 93.00 |  | 1918 |  | 1918 |  |
|  | | | | | | | |
