# Supplementary material for: Predicting survival from colorectal cancer histology slides using deep learning: A retrospective multicenter study
Source: PLoS Med. 2019 Jan 24;16(1):e1002730. doi: 10.1371/journal.pmed.1002730 (PMC6345440; doi:10.1371/journal.pmed.1002730)
Supplement: S6 Table — (DOCX) [file pmed.1002730.s012.docx]

|  | **Layer name** | **Layer type** | **Layer description** |
| --- | --- | --- | --- |
| 1 | input | Image Input | 224x224x3 images with 'zerocenter'normalization |
| 2 | conv1_1 | Convolution | 64 3x3x3 convolutions with stride [1 1] and padding [1 1 1 1] |
| 3 | relu1_1 | ReLU | ReLU |
| 4 | conv1_2 | Convolution | 64 3x3x64 convolutions with stride [1 1] and padding [1 1 1 1] |
| 5 | relu1_2 | ReLU | ReLU |
| 6 | pool1 | Max Pooling | 2x2 max pooling with stride [2 2] and padding [0 0 0 0] |
| 7 | conv2_1 | Convolution | 128 3x3x64 convolutions with stride [1 1] and padding [1 1 1 1] |
| 8 | relu2_1 | ReLU | ReLU |
| 9 | conv2_2 | Convolution | 128 3x3x128 convolutions with stride [1 1] and padding [1 1 1 1] |
| 10 | relu2_2 | ReLU | ReLU |
| 11 | pool2 | Max Pooling | 2x2 max pooling with stride [2 2] and padding [0 0 0 0] |
| 12 | conv3_1 | Convolution | 256 3x3x128 convolutions with stride [1 1] and padding [1 1 1 1] |
| 13 | relu3_1 | ReLU | ReLU |
| 14 | conv3_2 | Convolution | 256 3x3x256 convolutions with stride [1 1] and padding [1 1 1 1] |
| 15 | relu3_2 | ReLU | ReLU |
| 16 | conv3_3 | Convolution | 256 3x3x256 convolutions with stride [1 1] and padding [1 1 1 1] |
| 17 | relu3_3 | ReLU | ReLU |
| 18 | conv3_4 | Convolution | 256 3x3x256 convolutions with stride [1 1] and padding [1 1 1 1] |
| 19 | relu3_4 | ReLU | ReLU |
| 20 | pool3 | Max Pooling | 2x2 max pooling with stride [2 2] and padding [0 0 0 0] |
| 21 | conv4_1 | Convolution | 512 3x3x256 convolutions with stride [1 1] and padding [1 1 1 1] |
| 22 | relu4_1 | ReLU | ReLU |
| 23 | conv4_2 | Convolution | 512 3x3x512 convolutions with stride [1 1] and padding [1 1 1 1] |
| 24 | relu4_2 | ReLU | ReLU |
| 25 | conv4_3 | Convolution | 512 3x3x512 convolutions with stride [1 1] and padding [1 1 1 1] |
| 26 | relu4_3 | ReLU | ReLU |
| 27 | conv4_4 | Convolution | 512 3x3x512 convolutions with stride [1 1] and padding [1 1 1 1] |
| 28 | relu4_4 | ReLU | ReLU |
| 29 | pool4 | Max Pooling | 2x2 max pooling with stride [2 2] and padding [0 0 0 0] |
| 30 | conv5_1 | Convolution | 512 3x3x512 convolutions with stride [1 1] and padding [1 1 1 1] |
| 31 | relu5_1 | ReLU | ReLU |
| 32 | conv5_2 | Convolution | 512 3x3x512 convolutions with stride [1 1] and padding [1 1 1 1] |
| 33 | relu5_2 | ReLU | ReLU |
| 34 | conv5_3 | Convolution | 512 3x3x512 convolutions with stride [1 1] and padding [1 1 1 1] |
| 35 | relu5_3 | ReLU | ReLU |
| 36 | conv5_4 | Convolution | 512 3x3x512 convolutions with stride [1 1] and padding [1 1 1 1] |
| 37 | relu5_4 | ReLU | ReLU |
| 38 | pool5 | Max Pooling | 2x2 max pooling with stride [2 2] and padding [0 0 0 0] |
| 39 | fc6 | Fully Connected | 4096 fully connected layer |
| 40 | relu6 | ReLU | ReLU |
| 41 | drop6 | Dropout | 50% dropout |
| 42 | fc7 | Fully Connected | 4096 fully connected layer |
| 43 | relu7 | ReLU | ReLU |
| 44 | drop7 | Dropout | 50% dropout |
| 45 | fc | Fully Connected | 9 fully connected layer |
| 46 | prob | Softmax | softmax |
| 47 | classoutput | Classification | crossentropyex with 9 tissue classes |

**Suppl. Table 6: All layers in the final modified VGG19 convolutional neural network model**
