# Supplementary material for: Predicting survival from colorectal cancer histology slides using deep learning: A retrospective multicenter study
Source: PLoS Med. 2019 Jan 24;16(1):e1002730. doi: 10.1371/journal.pmed.1002730 (PMC6345440; doi:10.1371/journal.pmed.1002730)
Supplement: S8 Table — AUC, sensitivity, specificity, PPV, and NPV are shown as median with the 5th and 95th percentile of their distribution based on k = 25 random splits of the external validation set as shown in S6 Fig. AUC, area under the curve; CI, confidence interval; NPV, negative predictive value; PPV, positive predictive value. (DOCX) [file pmed.1002730.s014.docx]

|  | **AUC** | **Sensitivity** | **Specificity** | **PPV** | **NPV** |
| --- | --- | --- | --- | --- | --- |
| **ADI** | 1.00 [1.00, 1.00] | 1.00 [0.98, 1.00] | 1.00 [0.99, 1.00] | 1.00 [0.97, 1.00] | 1.00 [1.00, 1.00] |
| **BACK** | 1.00 [1.00, 1.00] | 1.00 [1.00, 1.00] | 1.00 [1.00, 1.00] | 1.00 [1.00, 1.00] | 1.00 [1.00, 1.00] |
| **DEB** | 1.00 [0.98, 1.00] | 1.00 [0.87, 1.00] | 0.99 [0.98, 0.99] | 0.80 [0.66, 0.88] | 1.00 [0.99, 1.00] |
| **LYM** | 1.00 [1.00, 1.00] | 0.89 [0.76, 0.94] | 1.00 [1.00, 1.00] | 1.00 [0.95, 1.00] | 0.99 [0.98, 0.99] |
| **MUC** | 1.00 [0.99, 1.00] | 0.98 [0.94, 1.00] | 0.99 [0.97, 1.00] | 0.92 [0.82, 0.98] | 1.00 [0.99, 1.00] |
| **MUS** | 0.98 [0.93, 1.00] | 0.90 [0.71, 0.98] | 0.99 [0.97, 1.00] | 0.88 [0.73, 0.97] | 0.99 [0.97, 1.00] |
| **NORM** | 1.00 [1.00, 1.00] | 0.97 [0.87, 1.00] | 0.99 [0.98, 1.00] | 0.93 [0.86, 1.00] | 1.00 [0.99, 1.00] |
| **STR** | 0.98 [0.97, 1.00] | 0.59 [0.43, 0.81] | 1.00 [0.99, 1.00] | 1.00 [0.83, 1.00] | 0.98 [0.95, 0.99] |
| **TUM** | 1.00 [1.00, 1.00] | 0.98 [0.93, 1.00] | 0.99 [0.98, 1.00] | 0.96 [0.90, 0.99] | 1.00 [0.98, 1.00] |

**Suppl. Table 8: Statistics for each tissue class in an external validation set.**
